# Ratiometric Detection of ATP by Fluorescent Cyclophanes with Bellows‐Type Sensing Mechanism

**DOI:** 10.1002/chem.202001523

**Published:** 2020-07-16

**Authors:** Aleksandr M. Agafontsev, Tatiana A. Shumilova, Aleksandr S. Oshchepkov, Frank Hampel, Evgeny A. Kataev

**Affiliations:** ^1^ N. N. Vorozhtsov Institute of Organic Chemistry SB RAS 9^th^ Lavrentiev Avenue 630090 Novosibirsk Russian Federation; ^2^ Institute of Chemistry Technische Universität Chemnitz 09107 Chemnitz Germany; ^3^ Department of Chemistry and Pharmacy University of Erlangen-Nürnberg Nikolaus-Fiebiger-Str. 10 91058 Erlangen Germany

**Keywords:** cyclophane, fluorescence sensing, host-guest chemistry, macrocycle, nucleotide recognition

## Abstract

Pyrene‐based cyclophanes have been synthesized with the aim to realize a bellows‐type sensing mechanism for the ratiometric detection of nucleotide concentrations in a buffered aqueous solution. The sensing mechanism involves the encapsulation of a nucleobase between two pyrene rings, which affects the monomer‐excimer equilibrium of the receptor in the excited state. The nature of the spacer and its connection pattern to pyrene rings have been varied to achieve high selectivity for ATP. The 1,8‐substituted pyrene‐based cyclophane with the 2,2’‐diaminodiethylamine spacer demonstrates the best selectivity for ATP showing a 50‐fold increase in the monomer‐excimer emission ratio upon saturation with the nucleotide. The receptor can detect ATP within the biological concentrations range over a wide pH range. NMR and spectroscopic studies have revealed the importance of hydrogen bonding and stacking interactions for achieving a required receptor selectivity. The probe has been successfully applied for the real‐time monitoring of creatine kinase activity.

## Graphical abstract

**Bellows‐type sensing mechanism**: the effect of amine spacers in pyrene‐based cyclophanes has been studied to reveal best structures for ratiometic detection of ATP through a guest‐dependent monomer‐excimer equilibrium.

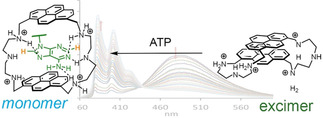

## Introduction

Nucleotides belong to an important class of phosphate derivatives, which are widespread in living systems and have various cellular functions. Nucleoside phosphates act as second messengers and serve as phosphate donors in kinase‐catalyzed protein phosphorylation.^1^ They are constituents of DNA and RNA structures, and thus can be targeted to affect genetic information transfer. ATP has been the most attractive target for recognition and detection because it plays an indispensable role in cell energy conversion in almost all metabolic cycles of living organisms. Cellular ATP concentration is maintained in the range of 1 to 10 mm.^2^ Biosensors such as luciferin‐luciferase bioluminescence assay, green fluorescent protein‐based probes and electrochemical sensors belong to the established methods of detection.^3^ However, small molecule‐based fluorescent probes are highly attractive in terms of their straightforward synthesis, high chemical stability and versatility in applications. Therefore, the development of artificial receptors and probes for nucleotides has attracted considerable attention in recent years.^2^, ^4^ In spite of much effort in this field, there is still a challenge to design a receptor that a) possesses high specificity for a certain nucleobase, b) can effectively work in an aqueous solution and c) generates a selective analytical signal after complex formation. Especially challenging has been the design of a signal transduction pathway from a nucleobase recognition event to an analytical signal.^5^

The analysis of ATP‐binding enzymes shows that nature has developed multiple ways for selective recognition of ATP. They include several sequence segments, where for example, glycine‐rich ones are responsible for phosphate binding and other segments bind to adenine.^6^ Adenine is often found in a hydrophobic cavity formed by lipophilic groups or aromatic rings that provide stacking interactions.^7^

The evidence of adenine‐arene interactions has been observed in the majority of the best receptors and probes for ATP reported so far.^2^ Among them are the hosts based on functionalized dyes^8^ probes bearing boronic acid for sugar recognition,^9^ positively charged cyclophanes,^10^ polyammonium‐based receptors,^11^ probes with transition metal sites,^12^ indicator displacement approach^13^ and sensors with aggregation‐induced sensing mechanism.^14^

Cyclophanes with aromatic rings oriented parallel to each other offer a great possibility to study dispersion interactions with a bound nucleobase. Fluorescent cyclophanes studied by us recently showed a turn‐on response for ATP; however, their selectivity was moderate. We found that anthracene‐based cyclophanes can intercalate with nucleobases between two dyes forming a sandwich‐like complex.^15^ This binding mode was reflected in an increase in the monomer emission band and a decrease in the excimer band. Interestingly, this is an opposite behavior of the anthracene‐based cyclophane described by Ramaiah and co‐workers,^16^ which showed a stabilization of the excimer form upon interaction with a guest under excitation. Guest‐induced changes in the excimer or monomer emission bands of pyrene‐containing hosts4f, 16, 17 have been observed in acyclic^18^ and macrocyclic receptors.18a, 19 However, the bellows‐type sensing mechanism, which involves stacking of a nucleobase between two pyrene rings of the cyclophane and changing the excimer‐monomer equilibrium, has not yet been realized.^20^ Towards the development of an ATP‐selective fluorescent receptor with bellows‐type sensing mechanism, we have proposed that the nature of the spacer between two pyrene rings and the connection pattern are decisive for the resulting receptor selectivity. Amine‐containing spacers should be protonated in aqueous solution leading to a repulsion of two pyrene rings. This repulsion should balance the attractive stacking interactions between rings and enable bellows‐type behavior of a cyclophane in the presence of an aromatic guest.

Herein, we report four pyrene‐based cyclophanes demonstrating a ratiometric fluorescence response towards nucleotides. The receptors possess a bellows‐type sensing mechanism, in which a nucleobase binds between two pyrene rings through intercalation with breaking of the preferred excimer complex in the excited state. We show that the amine spacer is important to achieve high selectivity for ATP. The 1,8‐substituted pyrene with the 2,2’‐diaminodiethylamine spacer (receptor **1**) demonstrates the highest selectivity for ATP showing a 50‐fold fluorescence ratio in the monomer‐excimer emission upon saturation with the nucleotide. The receptor can detect ATP within the biological concentrations range over a wide pH window. We also demonstrate that **1** functions as an efficient probe for the real‐time monitoring of creatine kinase activity. The reported binding mechanism offers also a strategy to synthesize new rotaxanes based on polyammonium cyclophanes.

## Results and Discussion

**Figure 1:**
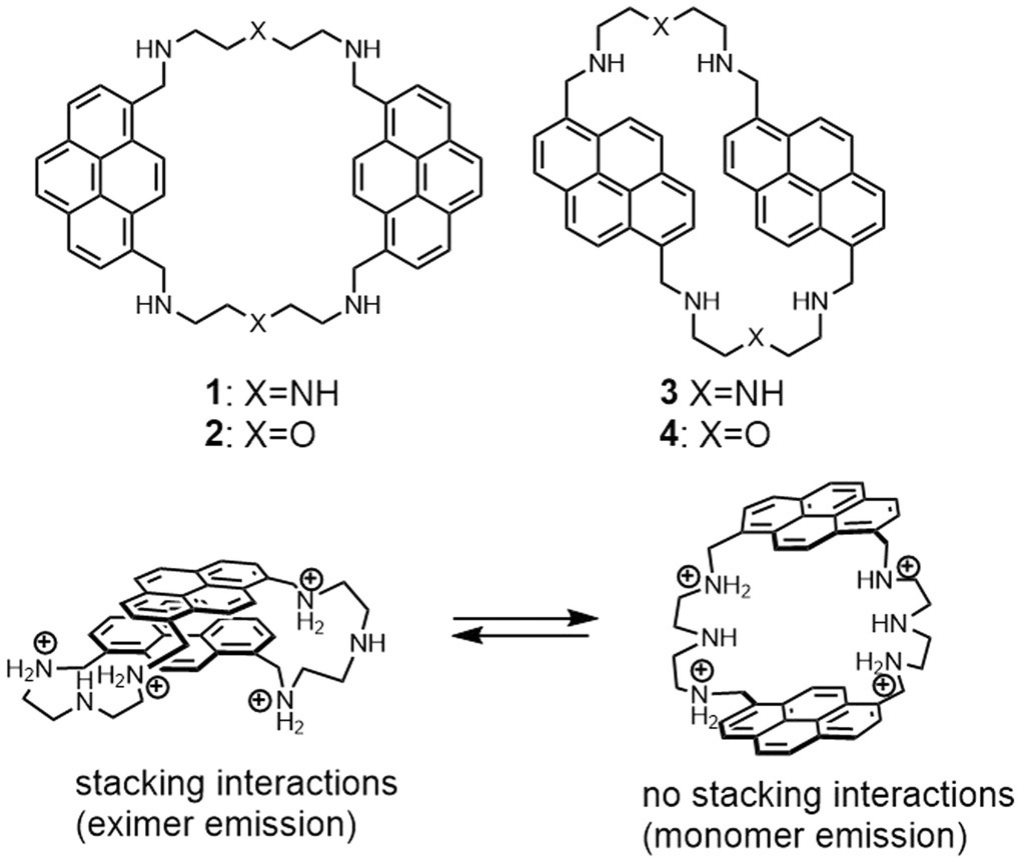
Structures of receptors **1**–**4** and conformational equilibrium of receptor **1** in aqueous solution, which describes the presence of the excimer and monomer emission in fluorescence spectra.

**Figure 2:**
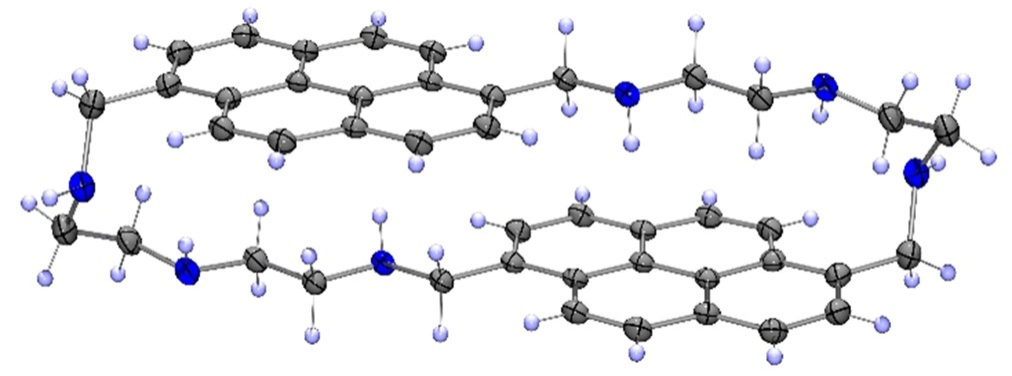
Single crystal structure of **3**. ORTEP‐rendered drawing with ellipsoids are shown at a 50 % probability level.

Macrocycles **1**–**4** (Figure 1) were synthesized with 30–60 % yields by a condensation of 2,2’‐diaminodiethylamine or 2‐(2‐aminoethoxy)ethylamine with 1,6‐ and 1,8 pyrenedicarboxaldehyde by an adapted method described by Fabbrizzi.^21^ The heteroatoms (NH, O) in the spacer structure were varied to understand if they could contribute to the stability and selectivity of the receptor. The same idea we pursued with two pyrene isomers, which produce macrocycles with different spatial ring orientation. Attempts to obtain single crystals were successful only for receptor **3**, because all the compounds tend to form amorphous powder in both organic and aqueous solution. Receptor **3** has an elongated conformation in the solid‐phase structure (Figure 2) and possesses intra‐ and intermolecular CH‐π interaction.

**Figure 3:**
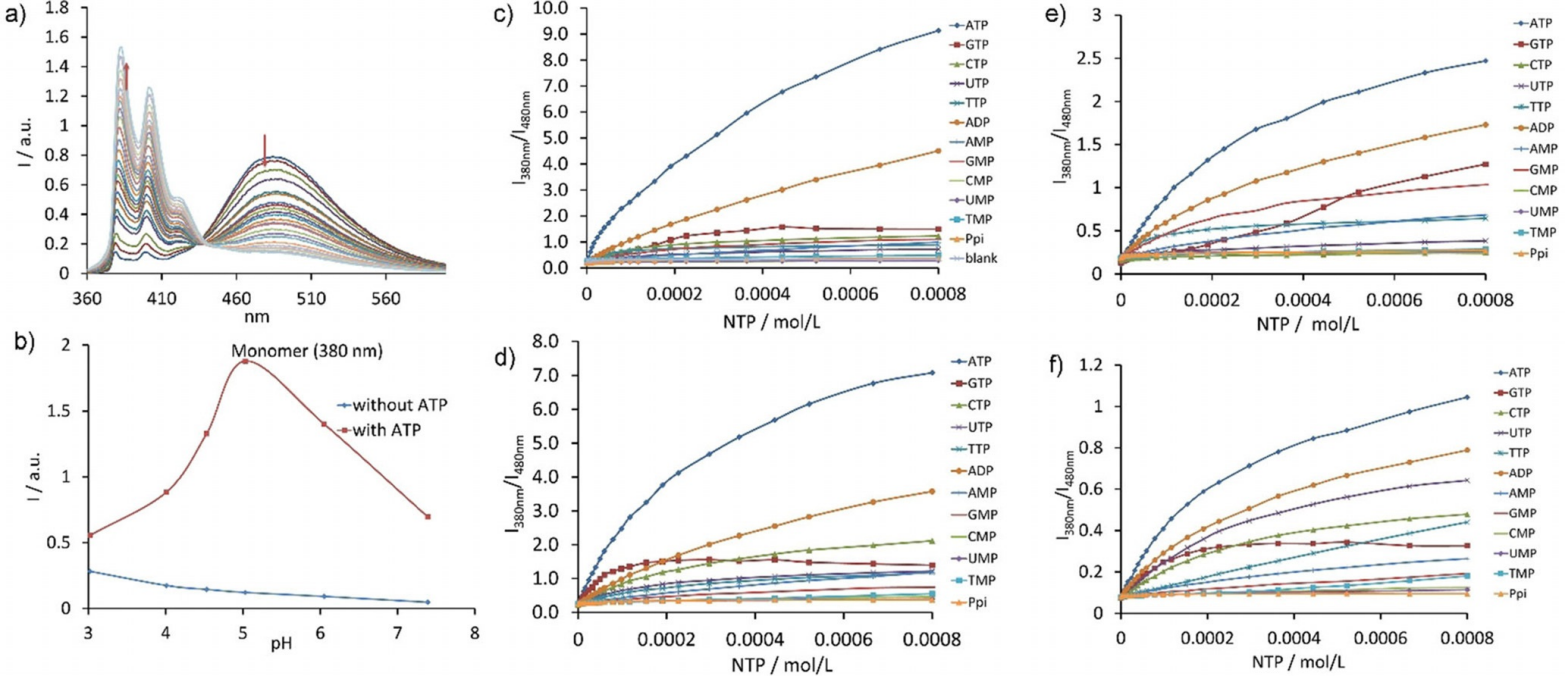
a) Fluorescence spectra of **1** during addition of increasing amounts of ATP. b) Changes in the monomer intensity band depending on the pH in the absence and in the presence of 10 equiv of ATP. Fluorescent enhancement observed for receptors c) **1**, d) **2**, e) **3**, and f) **4** upon addition of increasing amounts of nucleotides and pyrophosphate as a control anion. Conditions: 0.01 mm receptor **1**, λ_ex_=355 nm, 50 mm MES buffer (pH 6.2, 6 % DMSO), 25 °C. ATP—adenosine triphosphate; GTP—guanosine triphosphate; CTP—cytosine triphosphate; TTP—thymidine triphosphate; UTP—uridine triphosphate; NMP and NDP nucleotides, where N=A, T, U, C or G, correspond to mono‐ and diphosphates.

Initially, we determined the protonation constants of the receptors in water by using potentiometric titrations (stepwise log*K_a_* values are: 7.9; 7.2; 7.2; 6.0; 4.3; 4.1, Figure S7). The pH region 4–7 was most appropriate for binding studies, as receptors are present in this pH window in three‐ and fourfold protonated states.^22^ Fluorescence spectra of all receptors feature two emission bands at 380 and 480 nm, which correspond to the monomer and excimer emission bands (Figure 3 a). A series of experiments were conducted in order to determine which parameters have an effect on the monomer‐excimer equilibrium. At acidic conditions, the monomer form prevails, while at basic conditions the excimer form starts to dominate. The PET process from the amine groups to pyrene also contributes to fluorescence changes and is deactivated upon protonation (lowering the pH of the solution).^23^ Interestingly, the presence of ATP generates the strongest changes in emission bands in pH window 4–7 (Figure 3 b, Figure S8). Thus, we chose a closer to physiological pH buffer—a 50 mm MES buffer with pH 6.2 for the determination of binding constants. Small amount of DMSO in solution (6 % volume) was required to achieve good solubility of the receptor in aqueous solution. UV/Vis measurements at different receptor concentrations confirmed the absence of aggregation at 10^−5^ m and proved the fact that the excimer emission originates from intramolecular interactions in solution (Figure S14).

**Figure 4:**
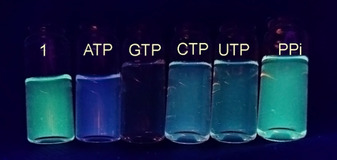
Changes in the emission colour of **1** in the presence of 100 equiv of NTPs.

Fluorescence titration experiments revealed that all nucleotides except GTP affect the monomer‐excimer equilibrium in solution and lead to an increase in the monomer emission band and a simultaneous decrease in the excimer band (*I*_380nm_/*I*_480nm_ reaches 10 for ATP). This fact supports the idea of the complex formation, in which a nucleobase is inserted between two pyrene rings. Contrary, GTP quenches the fluorescence of both bands with much less increment in the monomer emission (*I*_380nm_/*I*_480nm_ is approx. 2). Changes in the emission of for example, **1** after addition of excess of nucleotides can be easily seen with the naked eye (Figure 4). In cases of receptors **3** and **4** even a red shift (10 nm) of the excimer band is observed for both GTP and GMP nucleotides with small changes in the excimer‐monomer emission ratio indicating strong stacking interactions. The quenching effect of guanine derivatives has already been observed in the literature and was attributed to the PET process between guanine and pyrene.^24^

Fluorescence titrations were carried out with nucleoside triphosphates, selected di‐ and monophosphates, as well as with pyrophosphate as a control anion that does not bear a nucleobase. The analysis of the measured response revealed that **1** has the highest selectivity for ATP in terms of fluorescence enhancement and selectivity. As can be seen in Figure 3, plotting the ratio of two fluorescence bands against nucleotide concentration allows one to distinguish ATP from other nucleotides. Notably, both **1** and **2** bearing NH‐groups show a much better response (up to 10‐fold enhancement of the monomer emission) than that observed for **3** and **4** bearing the oxygen‐containing spacer (1.3–3 fold). Hence, we concluded that the oxygen‐containing spacer stabilizes the stacked conformation of receptors, while the amine spacer makes the system more labile due to the greater number of protonation sites. This fact is supported by slightly lower binding constants of **3** and **4** for nucleotides in comparison with those obtained for **1** and **2**. Analysis of Table 1 reveals that the receptors show similar affinity for ATP and ADP. This observation indicates that the charge of the anion does not considerably contribute to the overall affinity. As can be seen in Figure 3, receptor **1** has slightly better sensing selectivity than that of **2**. The monomer/excimer emission ratio (*I*_380nm_/*I*_480nm_) for receptor **1** reaches 50 and 23 upon saturation with ATP and ADP, respectively. Addition of other nucleotides does not exceed the value of 8. For receptor **2**, the *I*_380nm_/*I*_480nm_ for ATP is 30, while most of other nucleotides show 10‐fold changes. Receptors bind di‐ and triphosphates with a stepwise 1:1 and 1:2 binding mode as revealed from fitting analysis and Job plots. The data was fitted by HypSpec2014 computer program to obtain the binding constants.^25^ The 1:2 binding event likely originates from the fact that the receptor bears greater charge at chosen conditions than that of nucleotides^26^ or/and the ability of the second nucleobase to stack with pyrene rings from outside. The competition experiment for the detection of ATP in the presence of other nucleotides revealed that GTP has the strongest effect because GTP quenches almost completely the fluorescence of the receptor (Figure S10). We also tested the tolerance of the bellows‐type sensing mechanism towards the nature of the buffer and the pH of the solution. Fluorescence changes upon titration of **1** with ATP were similar; however the binding constants for ATP were slightly lower: log*K_11_*=4.50 in a 100 mm PBS (pH 7.4), log*K_11_*=4.33 in a 50 mm MOPSO (pH 6.2), log*K_11_*=4.46 in HEPES (pH 7.5). ITC measurements conducted for receptor **1** with ATP confirmed the results obtained by fluorescence and UV/Vis spectroscopy: log*K_11_*=5.61, Δ*H*=−86.4 kJ mol^−1^, *T*Δ*S*=−54.4 kJ mol^−1^ (Figure S16). Thus, recognition of ATP by the receptor is an enthalpically favorable and entropically unfavorable process.

**Table 1:** Binding constants of receptors **1**–**4** (log*K_11_*; log*K_12_*) for nucleotides and selected mono‐ and di‐phosphates obtained by fluorescence titrations in a 50 mm MES buffer (pH 6.2, 6 % DMSO). Excitation: 355 nm, slit 2/1, emission 360–600 nm.^[a]^

Nucleotide	**1**	**2**	**3**	**4**
ATP	5.48; 3.69	4.22; 3.73	5.55; 4.17	5.10; 3.52
GTP	4.38; 3.44	5.35; 3.68	5.35; 3.38	4.63; 4.29
CTP	4.26; 3.07	<1; 7.08	4.29; 3.25	3.94; <1
UTP	4.91; 3.42	3.20; 4.19	4.67; 3.28	3.97; <1
TTP	4.77; 3.41	4.35; 3.35	5.16; 3.64	2.72; <1
ADP	5.47; 3.70	4.44; 3.10	4.21; 3.00	5.00; 3.33
AMP	4.15	3.38	3.00	3.33
GMP	4.50	4.36	3.51	3.25

[a] Binding constants are an average values from 2–3 experiments with errors, which does not exceed 0.04 log units.

**Figure 5:**
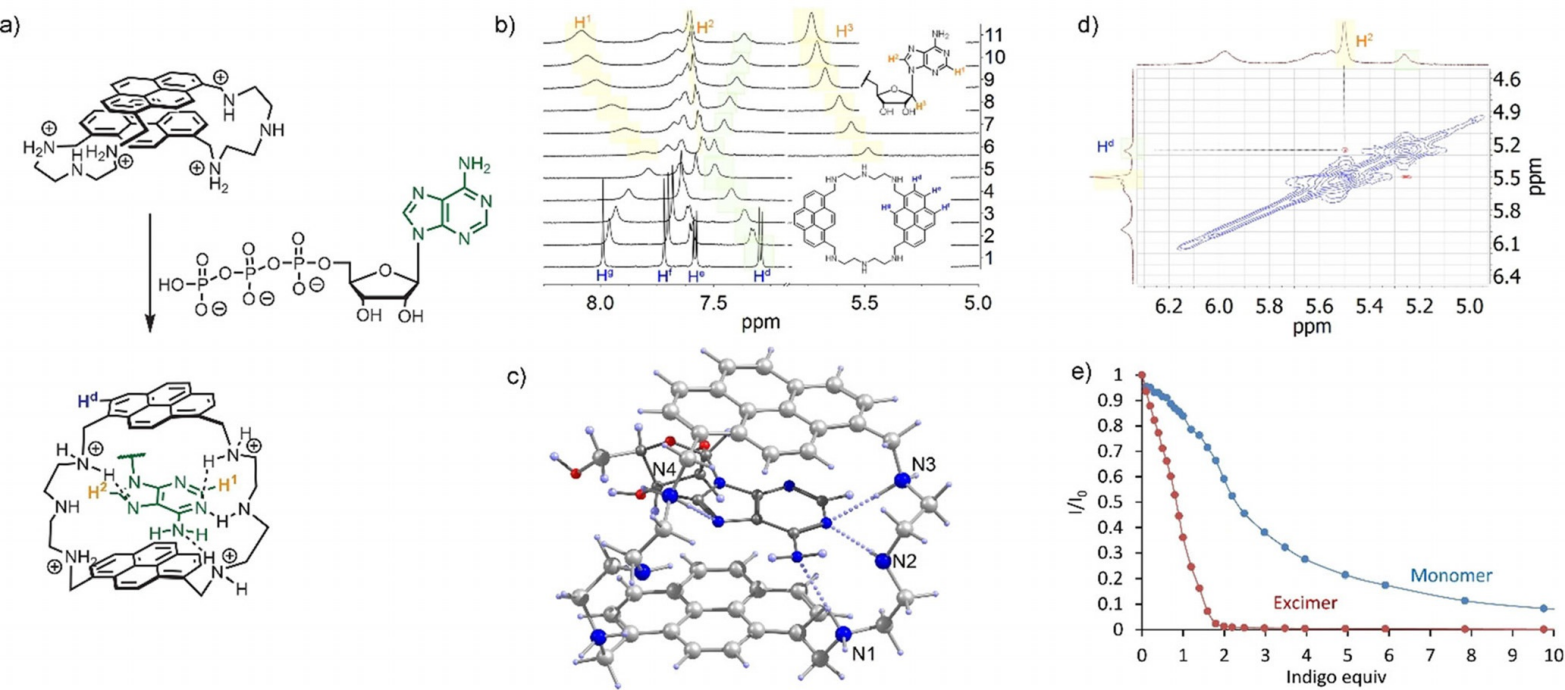
a) Structure of ATP complex according to DFT calculations and NMR studies. b) ^1^H NMR titration of **1** with AMP (0.5 mm receptor in a 50 mm MES, pH 6.2, 10 % [D_6_]DMSO). c) Optimized structure of the adenosine complex with receptor **1** according to MP2 calculations. d) ROESY spectrum of **1**AMP complex showing the cross signal between adenine and pyrene protons. e) Changes in the emission of excimer and monomer bands upon addition of indigo carmine to a solution of **1**.

To clarify the exact binding mode and structural parameters that are responsible for high ATP selectivity with receptor **1**, we conducted NMR studies together with DFT calculations of the host‐guest complex. The complexes of receptors with triphosphates were not soluble at concentrations higher than 10^−4^ M^.^ Therefore, we titrated receptor **1** with AMP in a 10 % [D_6_]DMSO‐buffer mixture (50 mm MES, pH 6.2 prepared in D_2_O). As can be inferred from Figure 5 b, the addition of AMP up to 1 equiv induces upfield shifts of most pyrene signals and downfield shifts of AMP signals. For the last mixture of **1** with 10 equiv of AMP we also obtained the ^1^H‐^1^H ROESY spectrum, which revealed a cross signal between adenine H^2^ proton and pyrene H^d^ proton (Figure 5 d). These results together with our spectroscopic studies provide solid evidence that the adenine ring intercalates with pyrene rings and break intramolecular pyrene‐pyrene stacking interactions in solution.

The origin of high ATP selectivity found for receptor **1** was further investigated by quantum chemical calculations. For simplicity reasons we used adenosine in calculations as a guest coordinated to the fourfold protonated receptor **1H_4_** ^**4+**^. Molecular geometries of complexes were optimized by a parametrized model involving dispersion interactions^27^ starting from a limited set of conformations from molecular dynamics.^28^ The geometry of the complex with the lowest energy was further optimized by MP2 method by using the L1 basis set (Figure 5 c).^29^ According to the calculations, the adenine ring intercalates with both pyrene rings and forms hydrogen bonds with the side chains—amine spacers. Protonated amines N1 and N3 form hydrogen bonds with the amine group and the pyrimidine nitrogen of the adenine ring. Interestingly, N4 forms a hydrogen bond with the adenine ring additionally stabilizing the overall complex. Thus, the combination of stacking interactions, complementary hydrogen bonding and appropriate position of hydrogen bond donor groups in space may be responsible for the observed selectivity for adenosine triphosphate.

The fact that our receptor can form sandwich complexes with aromatic guests was further evidenced by studying the fluorescence quenching of **1** by indigo carmine. Being an electron‐deficient π system, indigo carmine should strongly interact with electron‐rich pyrene. Especially strong interaction can be expected in the sandwich complex, which resembles the binding mode observed for the nucleotides. Since indigo absorbs light in the region where **1** emits, it should quench its fluorescence. Indeed, the fluorescence titration of **1** with up to 2 equiv indigo carmine led to a strong quenching of the excimer band (Figure 5 e). However, the monomer emission was not linearly quenched indicating a stepwise 1:1 and 1:2 complex formation with calculated binding constants log*K_11_*=5.67 and log*K_12_*=5.01. The observed difference in quenching supports our suggestion that indigo carmine deactivate the excimer complex and likely forms a complex, in which the dye stacks in a sequence pyrene‐indigo‐pyrene‐indigo. Such an alternate stacking mode was observed by us recently for large macrocycles with naphthalimide and pyridine rings.^30^

**Figure 6:**
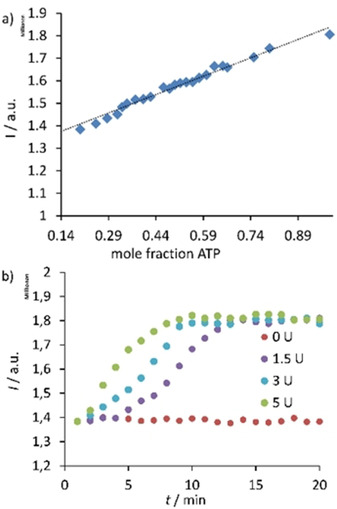
a) Fluorescence intensity of **1** at 380 nm versus mole fraction of ATP/ADP. ATP/ADP ratio was varied keeping the total nucleotide concentration constant (1 mm). b) Time‐dependent changes of fluorescence intensity at 383 nm during creatine dephosphorylation by creatine kinase (0–5 U). Conditions: 0.01 mm receptor, 1 mm ADP, 1 mm creatine phosphate, 50 mm MES buffer (pH 6.2), 0.1 % albumin, 25 °C, λ_ex_=355 nm.

The fact that receptor **1** can discriminate ATP from ADP and AMP encouraged us to investigate it as a probe in real‐time monitoring of enzymatic activity of creatine kinase. Clinical blood tests assay for creatine kinase are important for the detection of a series of diseases. Creatine kinase catalyzes the conversion of creatine phosphate to creatine and thus phosphorylates ADP to form ATP.^31^ We expected that fluorescence emission of **1** should be enhanced during this catalytic reaction. As it is shown in Figure 6 a, the plot of fluorescence at 380 nm (monomer emission) vs. mole fraction of ATP relative to ADP demonstrates a 1.5‐fold increase in the emission intensity. The time‐dependent measurements of creatine kinase activity revealed that the reaction rate increases in proportion to enzyme concentrations (Figure 6 b). The yield in the reaction of ATP formation can be assessed based on Figure 6 a. The observed fluorescence increase after the reaction was completed corresponds to the ATP mole fraction of approx. 0.9. Thus, receptor **1** represents a good probe for monitoring of creatine kinase activity.

## Conclusions

In summary, an ATP‐selective fluorescent receptor **1** that functions at pH 6.2 has been identified from a series of pyrene‐based cyclophanes. The receptor binds ATP with log*K_11_*=5.48 in a 50 mm MES buffer (pH 6.2) and shows a 50‐fold changes in in the monomer‐excimer emission ratio. The sensing mechanism involves the coordination of the adenine base between two pyrene rings, which subsequently leads to the breaking of the excimer complex in an aqueous solution. The observed decrease in the excimer emission and an increase in the monomer emission allow one to detect ATP ratiometrically in the biological concentration range. Quantum chemical calculation and detailed NMR studies show that high selectivity for ATP is achieved by an appropriate combination of π–π stacking pyrene‐adenine interactions with hydrogen bonding interactions between the adenine ring and the protonated amine spacer. The bellows‐type sensing mechanism allowed us to distinguish nucleotides from strongly competing pyrophosphate. A good level of selectivity for ATP over ADP, AMP and pyrophosphate enables the application of **1** in the monitoring of creatine kinase activity. Future work will focus on elucidating the ways to increase the selectivity of receptors by introduction of additional binding sites to the aromatic rings, as well as on exploring fluorescent cyclophanes to intercalate with DNA and RNA sequences.

## Experimental Section

Synthetic procedure, NMR, UV/Vis and fluorescent measurements, Cartesian coordinated of calculations, crystallographic data in CIF see the Supporting Information.

Deposition Number 1985471 contain(s) the supplementary crystallographic data for this paper. These data are provided free of charge by the joint Cambridge Crystallographic Data Centre and Fachinformationszentrum Karlsruhe Access Structures service www.ccdc.cam.ac.uk/structures.

## Conflict of interest

The authors declare no conflict of interest.

## Biographical Information

*Evgeny A. Kataev studied Chemistry at the M.V. Lomonosov Moscow State University (Diploma, 2003; Ph.D., 2006, Prof. Yuri A. Ustynyuk), University of Texas at Austin (Prof. Jonathan L. Sessler), EPFL Lausanne (Prof. Kay Severin) and University of Regensburg (Prof. Burkhard König). He began his independent research career at TU Chemnitz in 2011 as a Juniorprofessor and in 2017 was promoted to Apl. Professor. During 2019 he was interim professor at FAU Erlangen–Nürnberg and since April 2020 continued there as a Heisenberg Fellow of the DFG. The research activities of the group focus on the development of new concepts for supramolecular recognition and sensing in water, as well as on molecular switches*.

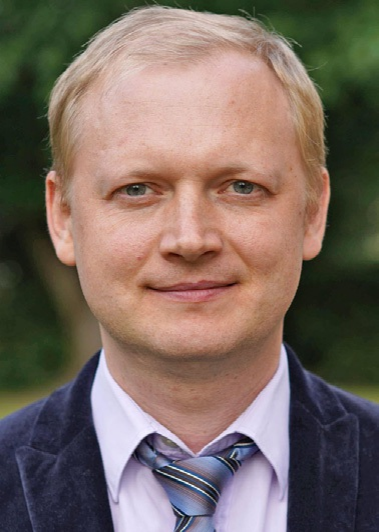

## Supporting information

As a service to our authors and readers, this journal provides supporting information supplied by the authors. Such materials are peer reviewed and may be re‐organized for online delivery, but are not copy‐edited or typeset. Technical support issues arising from supporting information (other than missing files) should be addressed to the authors.

Supplementary
