# Supplementary material for: Ratiometric Detection of ATP by Fluorescent Cyclophanes with Bellows‐Type Sensing Mechanism
Source: Chemistry. 2020 Jul 16;26(44):9991–7. doi: 10.1002/chem.202001523 (PMC7496914; doi:10.1002/chem.202001523)
Supplement: Supplementary file 1 — Supplementary [file CHEM-26-9991-s001.pdf]

Chemistry–A European Journal

Supporting Information

Ratiometric Detection of ATP by Fluorescent Cyclophanes with Bellows-Type Sensing Mechanism

Aleksandr M. Agafontsev,[a, b] Tatiana A. Shumilova,[b] Aleksandr S. Oshchepkov,[b] Frank Hampel,[c] and Evgeny A. Kataev*[c]

Supporting Information

Ratiometric detection of ATP by fluorescent cyclophanes with harmonica-like sensing mechanism

Aleksandr M. Agafontsev,[a,b] Tatiana A. Shumilova,[b] Aleksandr S. Oshchepkov,[b] Frank Hampel,[c] and Evgeny A. Kataev*[c]

[a] N.N. Vorozhtsov Institute of Organic Chemistry SB RAS, 9th Lavrentiev Avenue, 630090, Novosibirsk, Russian Federation
[b] Institute of Chemistry, Technische Universität Chemnitz, 09107 Chemnitz, Germany
[c] Department of Chemistry and Pharmacy, University Erlangen-Nürnberg, Nikolaus-Fiebiger-Str. 10, 91058 Erlangen, Germany

General

All the solvents were dried according to standard procedures. Reactions were performed in oven-dried round bottom flask. Crude products were purified by column chromatography on silica gel 100-200 mesh. TLC plates were visualized by exposure to ultraviolet light and/or by exposure to acidic ethanolic solution of ninhydrin followed by heating (<1 min) on a heat gun (~250 °C). Organic solutions were concentrated on rotary evaporator at 35–40 °C. **NMR Spectra**: Brucker Avance 600 Mhz. The chemical shifts are reported in δ [ppm] relative to external standards (solvent residual peak). The spectra were analyzed by first order, the coupling constants are given in Hertz [Hz]. Characterisation of the signals: s = singlet, d = doublet, t = triplet, q = quartet, m = multiplet, br = broad, dd = double doublet. Integration is determined as the relative number of atoms. The solvent used is reported for each spectrum. **Mass Spectra**: Finnigan MAT TSQ 7000 (ESI). **Melting Point**: Melting Points were determined on Büchi SMP or a Lambda PhotometricsOptiMelt MPA 100.

Synthesis of compounds

1,6-Dibromopyrene 1,8-dibromopyrene synthesized according to literature methods without modifications.[1] Already published procedures were used to synthesize pyrene-1,8-dicarbaldehyde or pyrene-1,6-dicarbaldehyde.[2]

The general conditions for the dialdehyde synthesis are the following: A solution of n-butyllithium in hexane (2.5M, 6 equiv) was added dropwise to a solution of a mixture of 1,6- and 1,8-dibromopyrene (1:1 molar ration obtained from the previous reaction) in anhydrous THF under an atmosphere of argon at –50°C. Reaction was stirred for 20 min at this temperature and then warmed to 25°C and stirred for additional 1 h. The reaction mixture was cooled to –40°C and DMF (6 equiv) was added dropwise to the solution. The mixture was stirred at room temperature for 12 h before water was added. THF was removed under reduced pressure. The resulting residue was suspended in 100 ml of H_2O and 200 ml DCM and the aqueous phase was extracted three times with 200 ml of CH_2Cl_2. The organic phases were combined and dried over $NaSO_4$. Solvents were removed under reduced pressure. The residue was purified by a column chromatography on silica gel using eluent CH_2Cl_2-hexane 1:1 with decreasing hexane portion to pure DCM. The first fraction contained pyrene-1,6-dicarbaldehyde (25% yield), the second fraction contained pyrene-1,8-dicarbaldehyde (21% yield).

The general method for the synthesis of pyrene-macrocycles

The synthesis of macrocyclic receptors was adapted from the method described by Fabbrizzi.[3] Pyrene-1,8-dicarbaldehyde or pyrene-1,6-dicarbaldehyde (3 mM, 774 mg) was added to a round bottom flask, then 400 ml of acetonitrile and 40 ml of methanol were added. The flask was placed in an oil bath and heated to 50°C with stirring, until the dialdehyde was completely dissolved. The appropriate amine (3 mmol) was dissolved in 100 ml of acetonitrile. The resulting solution was slowly added from the dropping funnel to the pyrene-1,8-dicarbaldehyde or pyrene-1,6-dicarbaldehyde solution with stirring and heating at 50°C. The reaction was achieved by heating at 50°C for 72 h. The solvent was removed under reduced pressure without heating. To the residue was added 300 ml of methanol and 30 mmoles of sodium borohydride, the flask was placed in an oil bath and heated to 50°C for 3 hours, then the reaction was kept overnight at room temperature. Methanol was removed under reduced pressure. The resulting solid was suspended in 100 ml of H_2O and 100 ml mixture chloroform-ethanol 10:100 and the aqueous phase was extracted with three portions 100 ml of mixture chloroform-ethanol 10:100. The organic phases were collected and dried with $NaSO_4$. Solvents were removed under reduced pressure. The residue was purified by chromatography on silica gel using eluent ethanol-chloroform 1:1 to ethanol-chloroform-ammonia 100:100:5.

6,16-dioxa-3,9,13,19-tetraaza-1,11(1,6)-dipyrenacycloicosaphane

The product is a pale yellow powder. Yield: 43%. M.p. 176-179^{0}C. ^{1}H NMR (600 MHz, $CDCl_3$) δ 7.96 (d, 4H), 7.57 (d, 4H), 7.48 (d. 4H), 7.38 (d, 4H), 4.33 (s, 8H), 3.76 (t, 8H), 3.10 (t, 8H). ^{13}C NMR (151 MHz, $CDCl_3$) δ 133.2, 129.8, 128.7, 127.4, 126.7, 124.6, 124.5, 121.9, 70.7, 52.2, 49.6. HRMS (ESI-TOF) m/z: $[M+H]^+$ calcd for $C_{44}H_{45}N_4O_2$, 661.3537, found 661.3541.

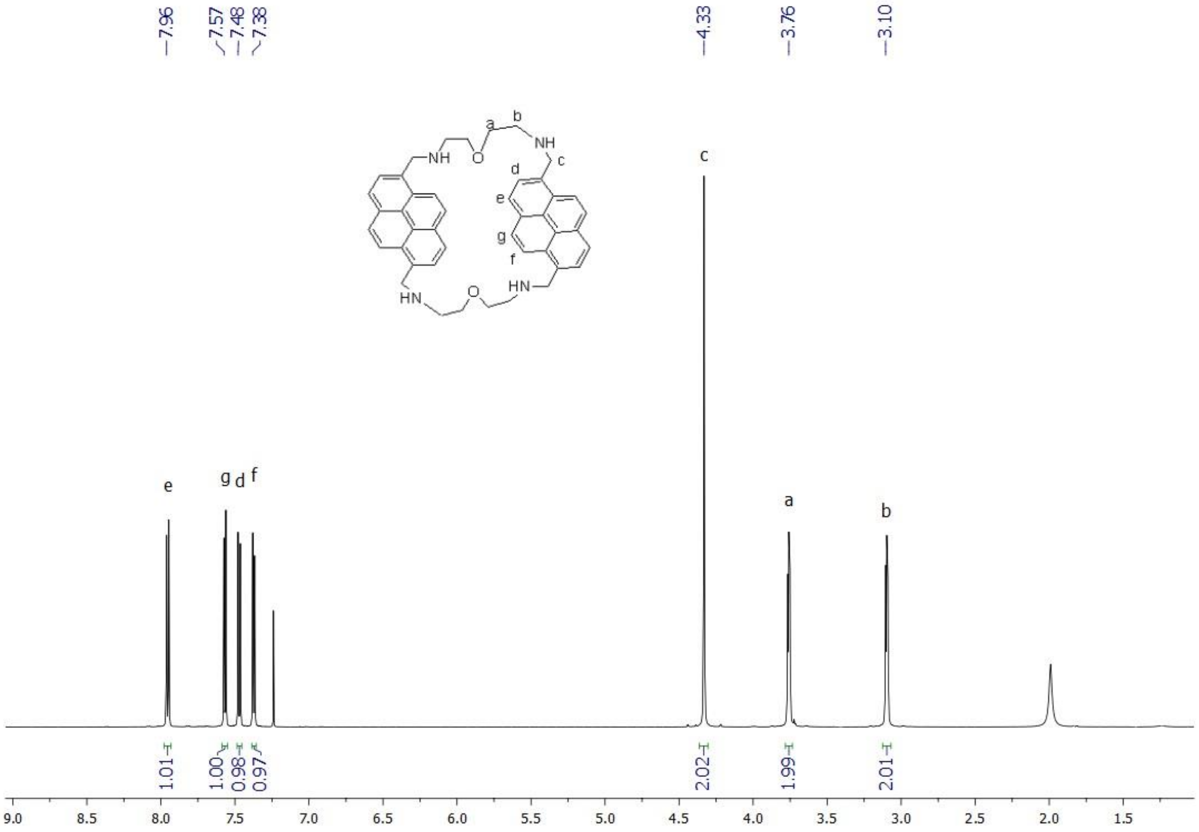

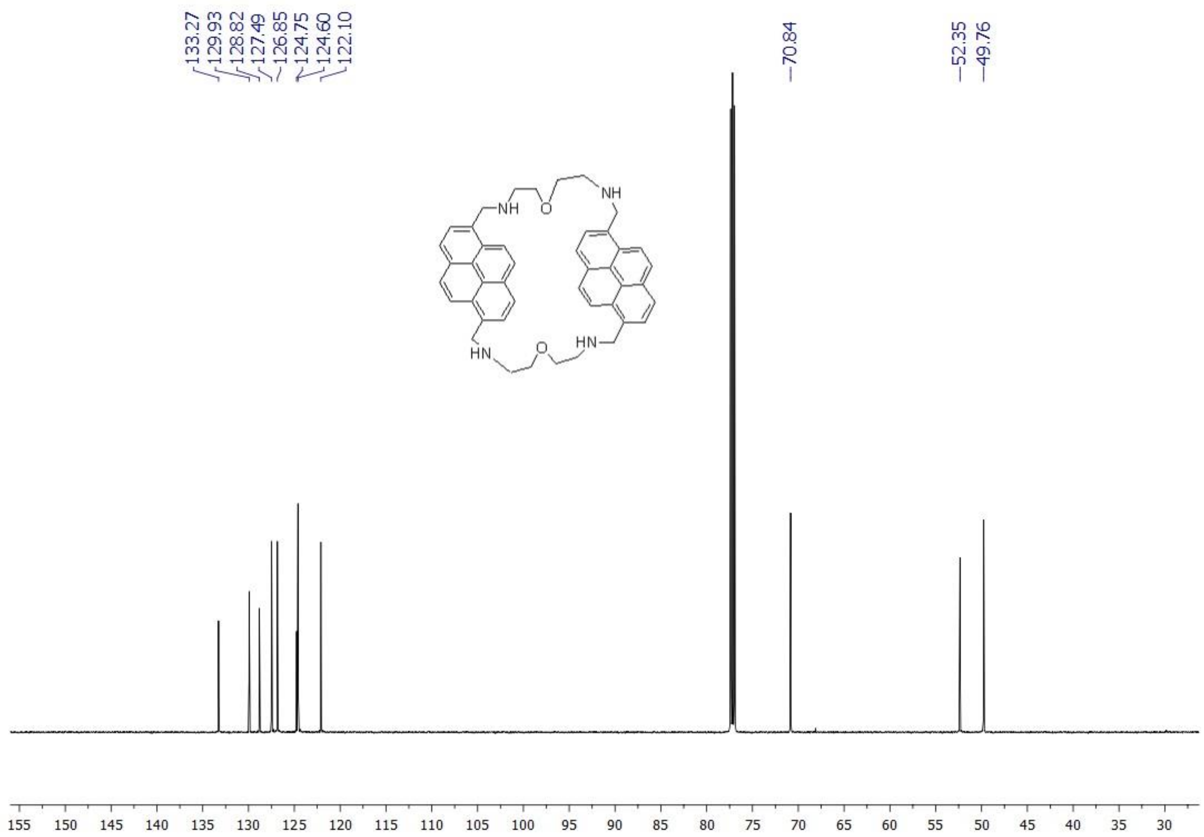

Figure S1. ^{1}H and ^{13}C NMR spectra of compound **4** measured in $CDCl_3$.

6,16-dioxa-3,9,13,19-tetraaza-1,11(1,8)-dipyrenacycloicosaphane

The product was obtained after chromatography as a pale yellow powder. Yield: 43%. M.p. 192-195^{0}C. ^{1}H NMR (600 MHz, CD_3OD, δ, ppm) 7.72 (s, 4H), 7.40 (s, 4H), 7.24 (m, 4H), 7.05 (d, 4H), 3.92 (s, 8H), 3.37 (t, 8H), 2.68 (t, 8H). ^{13}C NMR (151 MHz, CD_3OD) δ 131.2, 130.2, 127.3, 126.7, 125.8, 125.8, 124.3, 124.2, 122.1, 68.8, 50.2. HRMS (ESI-TOF) m/z: calcd for $C_{44}H_{45}N_4O_2$ $[M + H]^+$ 661.3537, found: m/z = 661.3539.

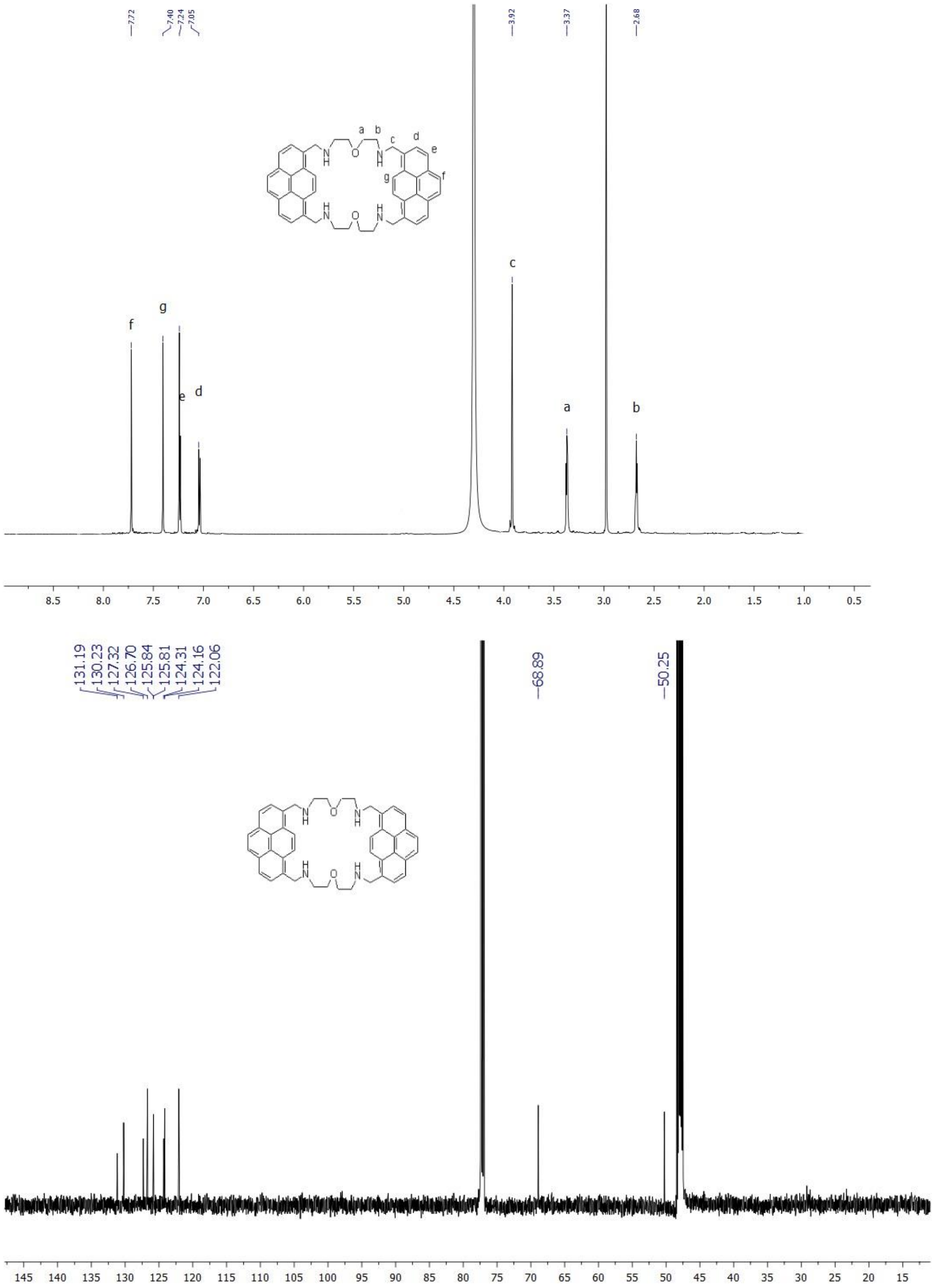

Figure S2. ^{1}H and ^{13}C NMR spectra of compound **2** measured in CD_3OD.

3,6,9,13,16,19-hexaaza-1,11(1,6)-dipyrenacycloicosaphane

The product was obtained after chromatography as a pale yellow powder. Yield: 56%. M.p. 180-183^{0}C. ^{1}H NMR (600 MHz, $CDCl_3$, δ, ppm) 8.15 (d, 4H), 7.65 (d, 4H), 7.62 (d, 4H), 7.48 (d, 4H), 4.28 (s, 8H), 2.95 (t, 8H), 2.76 (t, 8H). ^{13}C NMR (151 MHz, $CDCl_3$) δ 133.8, 130.2, 129.2, 127.5, 126.9, 125.1, 124.6, 122.8, 52.2, 49.5, 49.0. HRMS (ESI-TOF) m/z: calcd for $C_{44}H_{47}N_6$ $[M + H]^+$ 659.3856, found 659.3857.

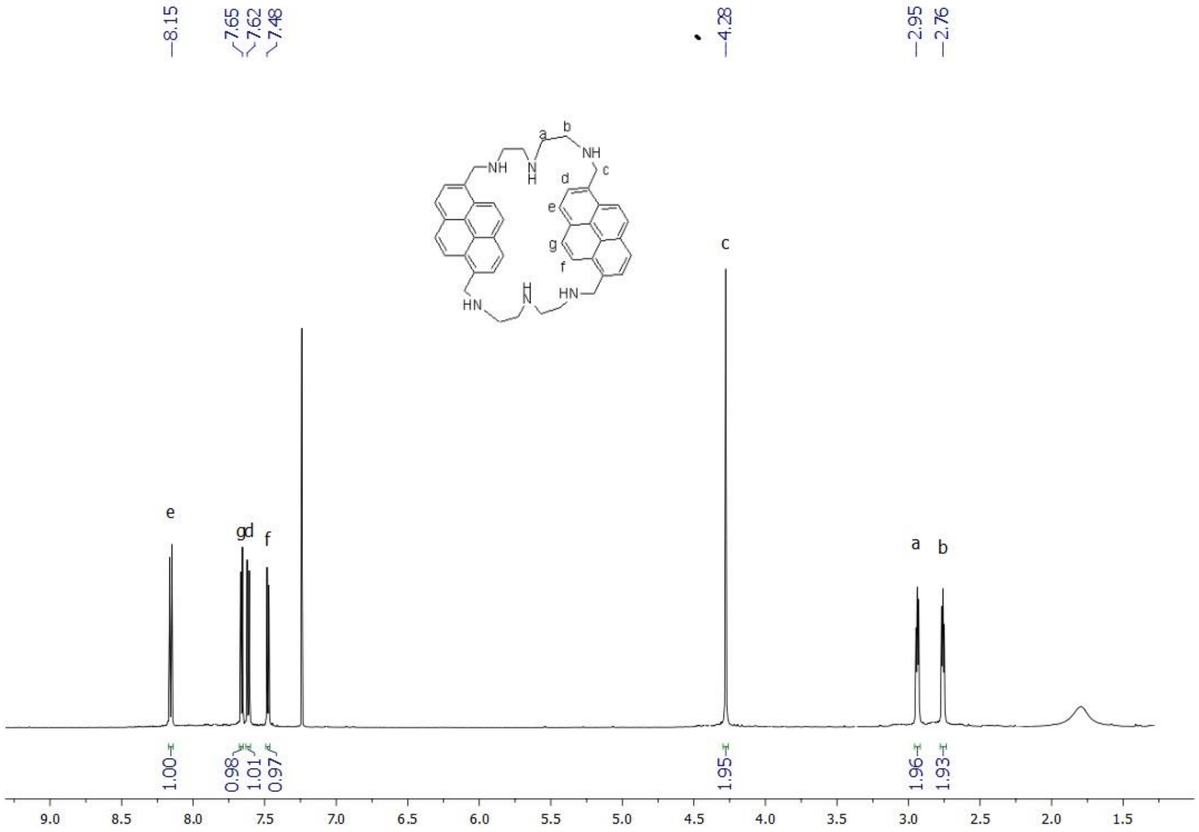

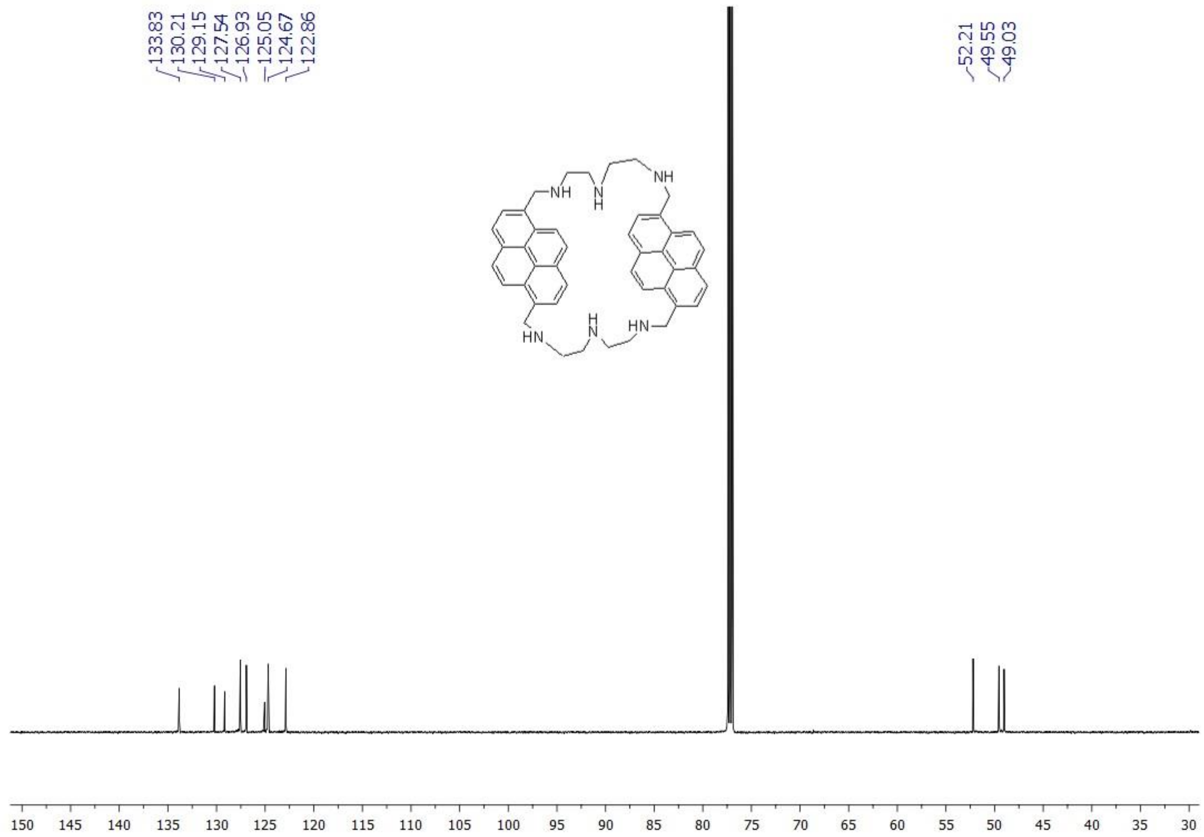

Figure S3. ^{1}H and ^{13}C NMR spectra of compound **3** measured in $CDCl_3$.

3,6,9,13,16,19-hexaaza-1,11(1,8)-dipyrenacycloicosaphane

The product was obtained after chromatography as a pale yellow powder. Yield: 29%. M.p. 158-161^{0}C. ^{1}H NMR (600 MHz, $CDCl_3$, δ, ppm) 8.25 (s, 4H), 7.82 (s, 4H), 7.79 (d, 4H), 7.72 (d. 4H), 4.25 (s, 8H), 2.79 (t, 8H), 2.65 (t, 8H). ^{13}C NMR (151 MHz, $CDCl_3$, δ, ppm) 133.8, 130.8, 128.6, 127.2, 126.7, 125.3, 124.7, 123.4, 51.8, 48.8, 48.7. HRMS (ESI-TOF) m/z: calcd for $C_{44}H_{47}N_6$ $[M + H]^+$ 659.3856, found 659.3859.

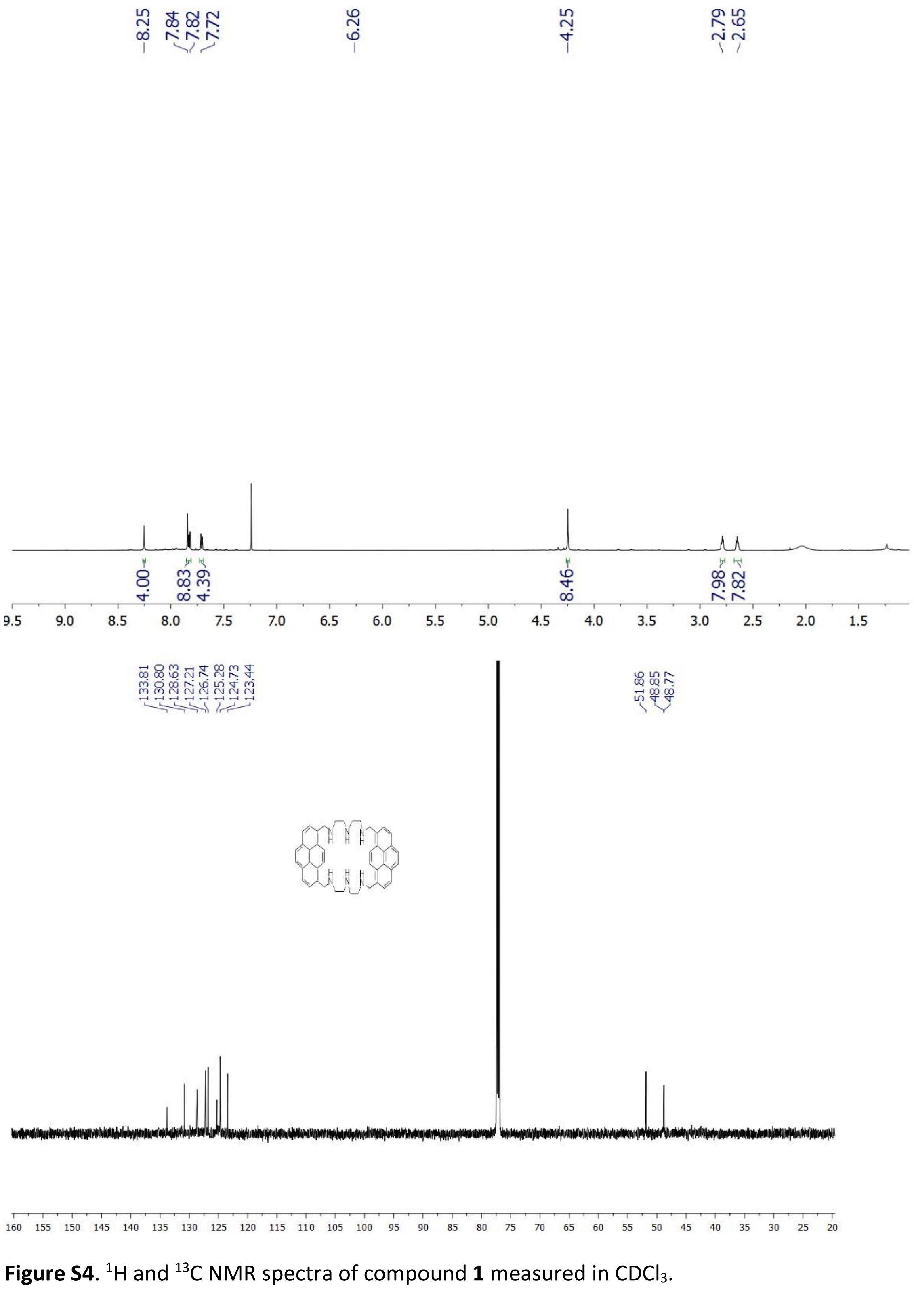

Figure S4. ^{1}H and ^{13}C NMR spectra of compound **1** measured in $CDCl_3$.

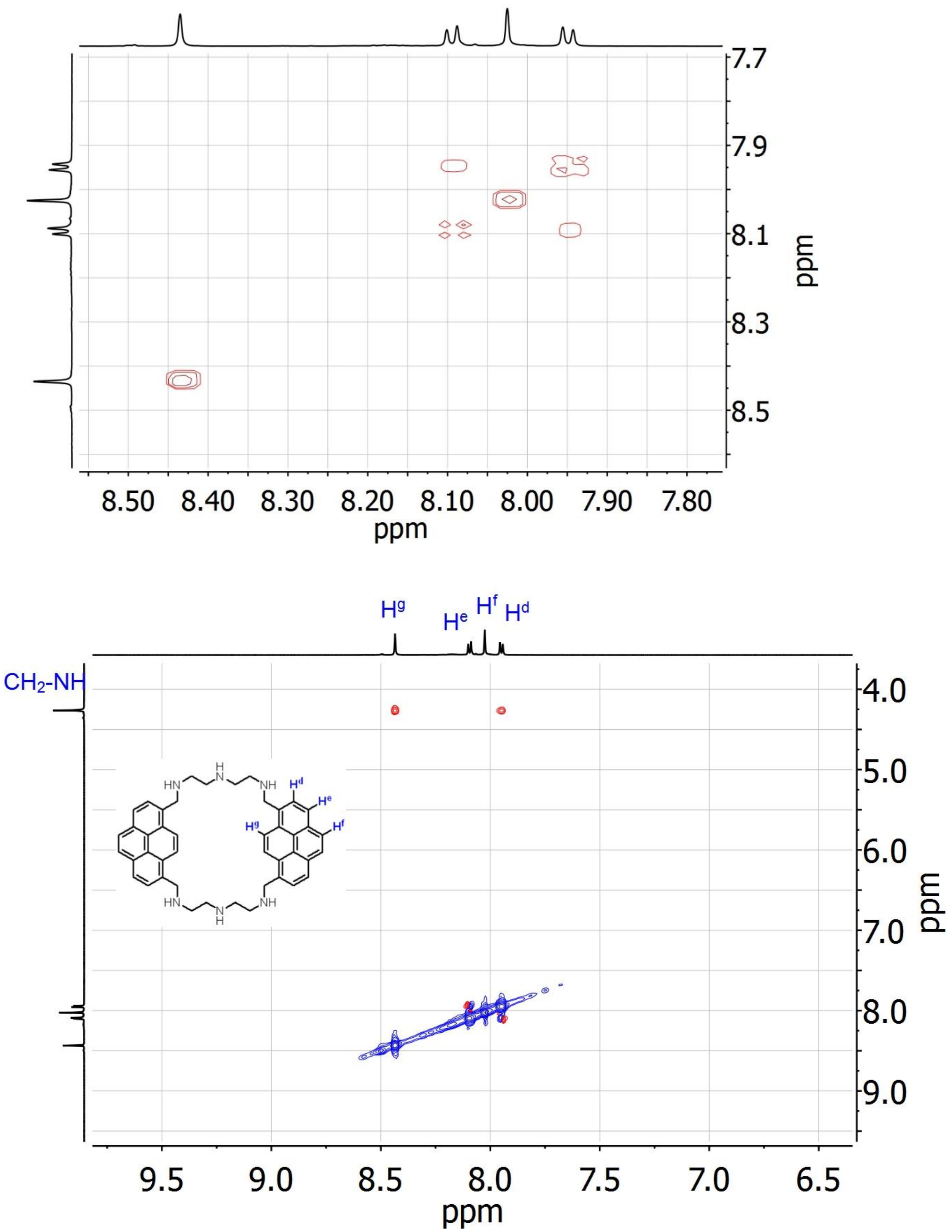

Figure S5. COSY and ROESY spectra of compound **1** measured in DMSO-*d6*.

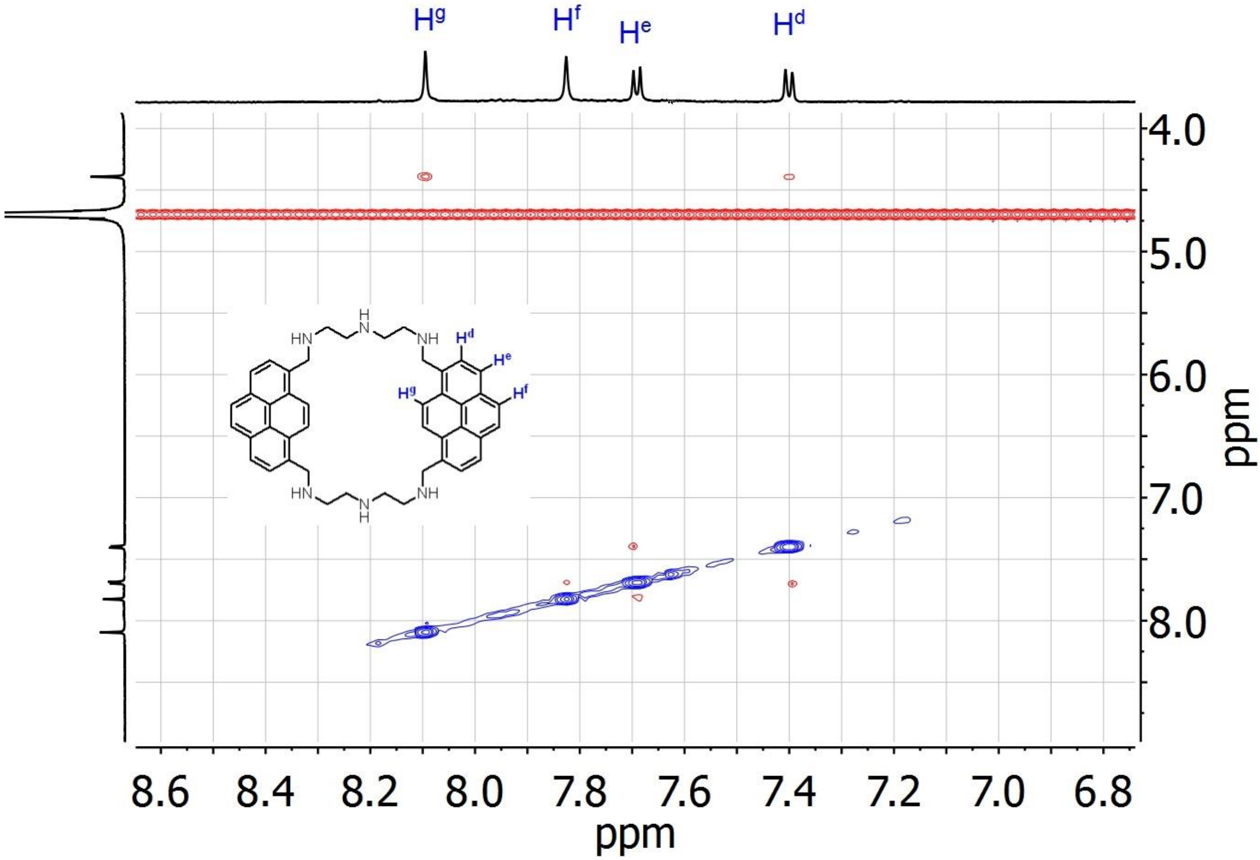

Figure S6. ROESY spectra of compound **1** measured in a 50 mM MES buffer pH 6.2.

Potentiometric studies of receptor 1.

All solutions were prepared in 0.05M NaCl solution with ca. 0.5 mM concentration of compounds in deionised water. For titrations standard 0.1M solution of NaOH was used. The potentiometric titrations were carried out on a Mettler Toledo G20 Titrator equipped with a DGi 102-Mini pH-electrode. The electrode was calibrated with standard calibrating solutions from Mettler Toledo. The reaction vessel was kept at constant temperature 23°C. The value of K'w was determined from data obtained in the alkaline range of the titration, and found to be equal to 13-14.00 in our experimental conditions. The titration experiment was carried out as follows: in the reaction vessel was placed a solution of a compound (and calculated amount of HCl); after stirring the solution for 5 minutes the titrations was started. The experiment was repeated 3-5 times. For the experiment in the presence of an anion, the corresponding amount of its solution was added prior to the titrations. The obtained data was imported to the HYPEQUAD 2008 program and fitted to obtained protonation constants.[4]

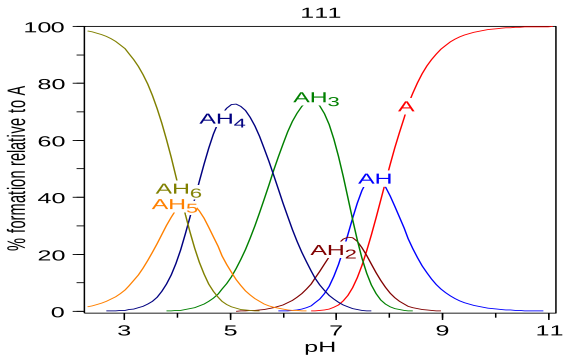

Figure S7. Distribution diagram of protonated forms of receptor **1** depending on the pH of the solution.

Fluorescence studies

Relationship between pH and fluorescence intensity

The solution with a desired pH value were prepared by adjusting 50 mM solution of acetic acid with an appropriate amount of sodium hydroxide. Receptor concentration 10^{-5} M was achieve by addition of a receptor in DMSO to an aqueous solution with the fixed pH. Content of DMSO in the final solution was 6% vol. Excitation at 350 nm, slit 2:1.

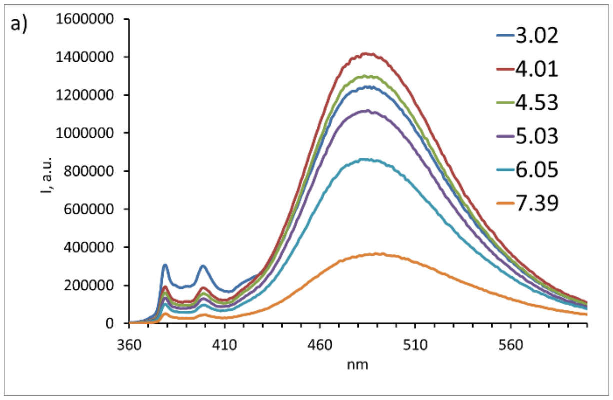

Figure S8. Fluorescence spectra of receptor **1** depending on the pH of the solution.

Competitive binding of ATP

The competition experiment was conducted as follows: 10^{-5} M solution of the receptor in a buffered solution (50 mM MES, pH 6.2, 6% DMSO) was treated first with excess ATP (50 equiv) dissolved in the same buffer and fluorescence was measured before and after addition. The ratio of intensities (I/I_0) represents the relative fluorescence increase after addition of ATP (marked and “None” below in Figure). The following experiments were done with a competing nucleotide and a mixture of a competing nucleotide with ATP. For example, the fluorescence the receptor was measured a) in the presence of 100 equiv of GTP and b) in the presence of 100 equiv of GTP together with 50 equiv of ATP (see Figure below).

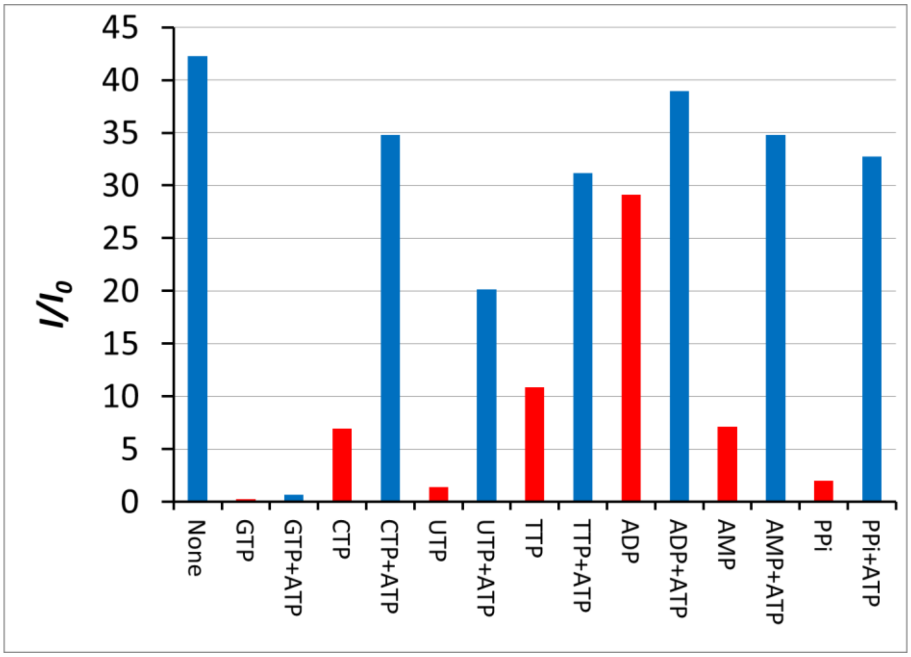

Figure S9. Competitive experiment for receptor **1** for ATP detection in the presence of other nucleotides. “None” corresponds to fluorescence changes after addition of 50 equiv of ATP. “GTP” corresponds to fluorescence changes after addition of 100 equiv. of GTP. “GTP+ATP” corresponds to fluorescence changes after addition 100 equiv. of GTP and 50 equiv. of ATP.

Fluorescence titration of receptors with nucleotides

The receptors (0.01 mM) were dissolved in a buffered solution (50mM MES, pH 6.2, 6%DMSO) and then titrated with nucleotides (0.02M) followed by fluorescence measurements of each titration point.

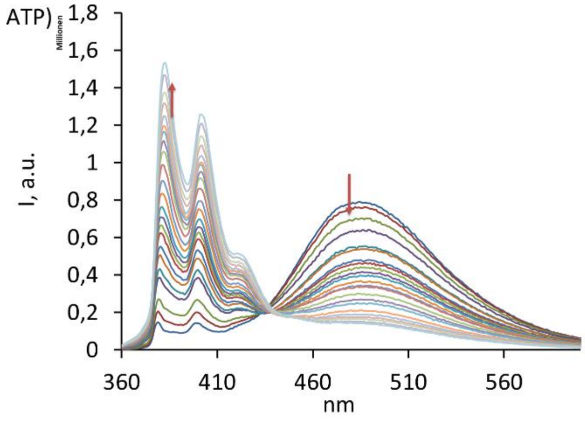

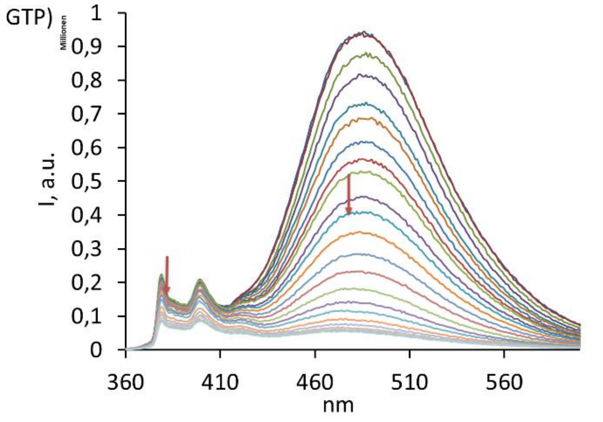

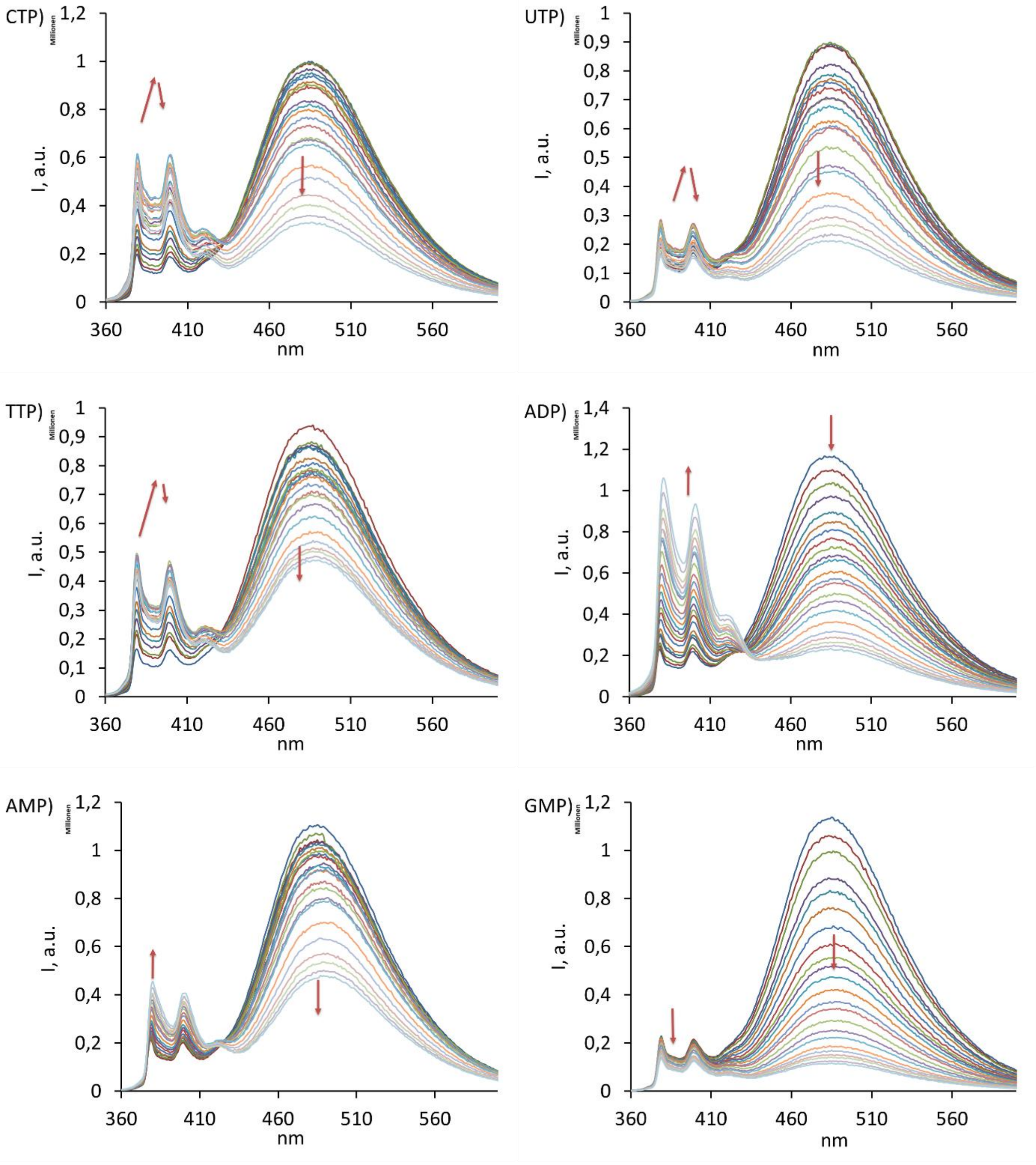

Figure S10. Fluorescence changes for **1** induced by addition of NTPs and other nucleoside mono- and diphosphates.

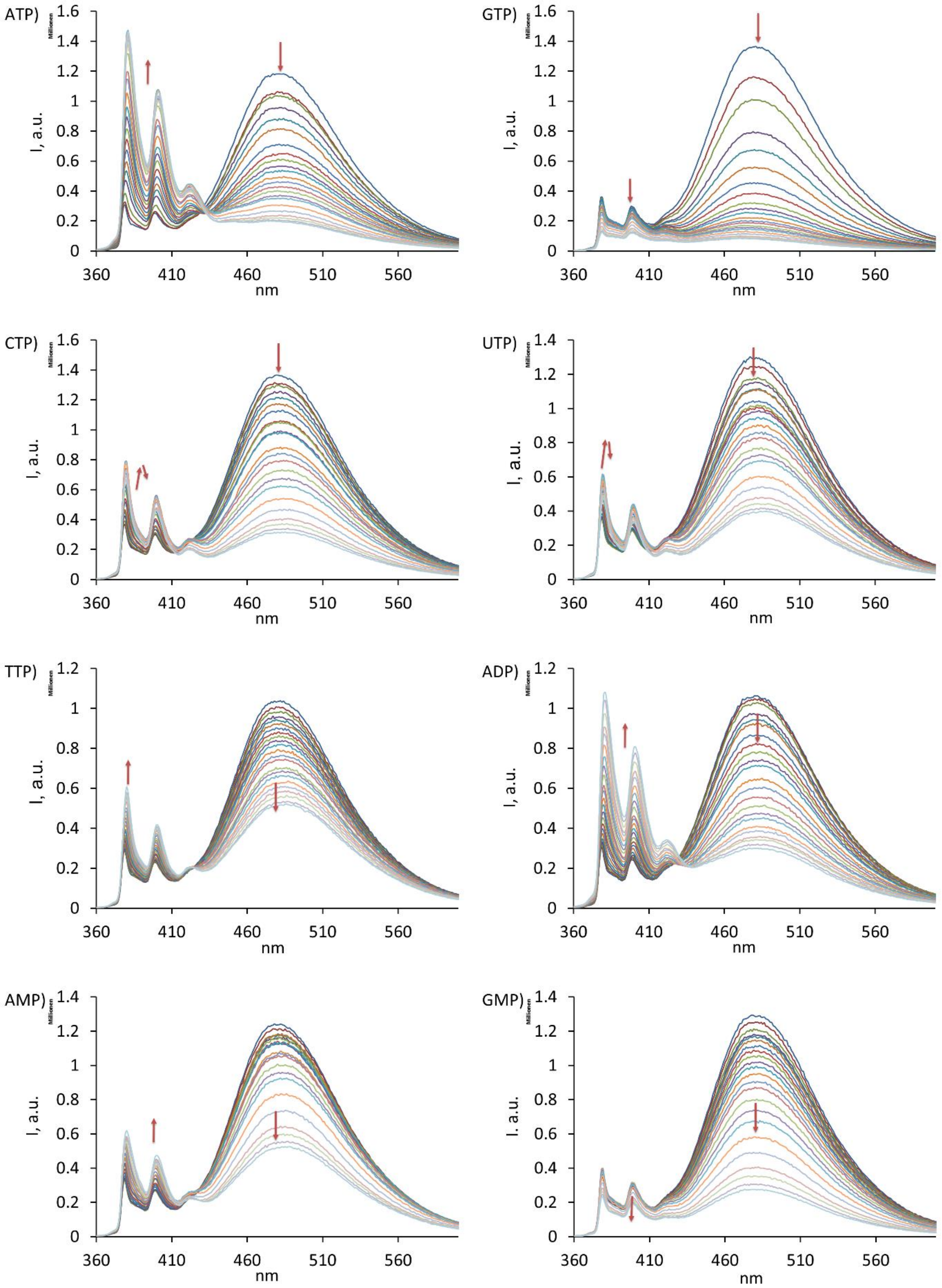

Figure S11. Fluorescence changes for **3** induced by addition of NTPs and other nucleoside mono- and diphosphates.

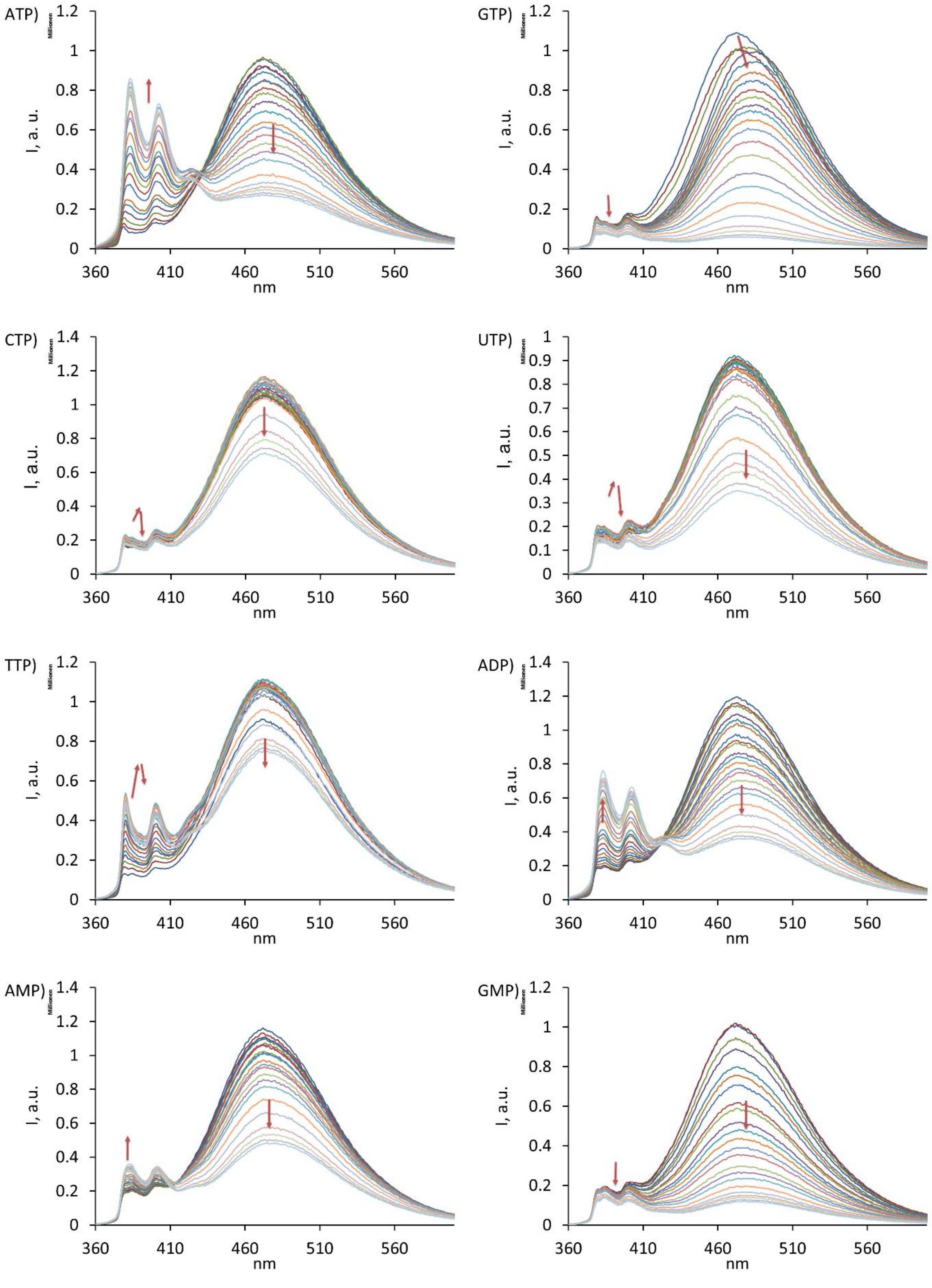

Figure S12. Fluorescence changes for **2** induced by addition of NTPs and other nucleoside mono- and diphosphates.

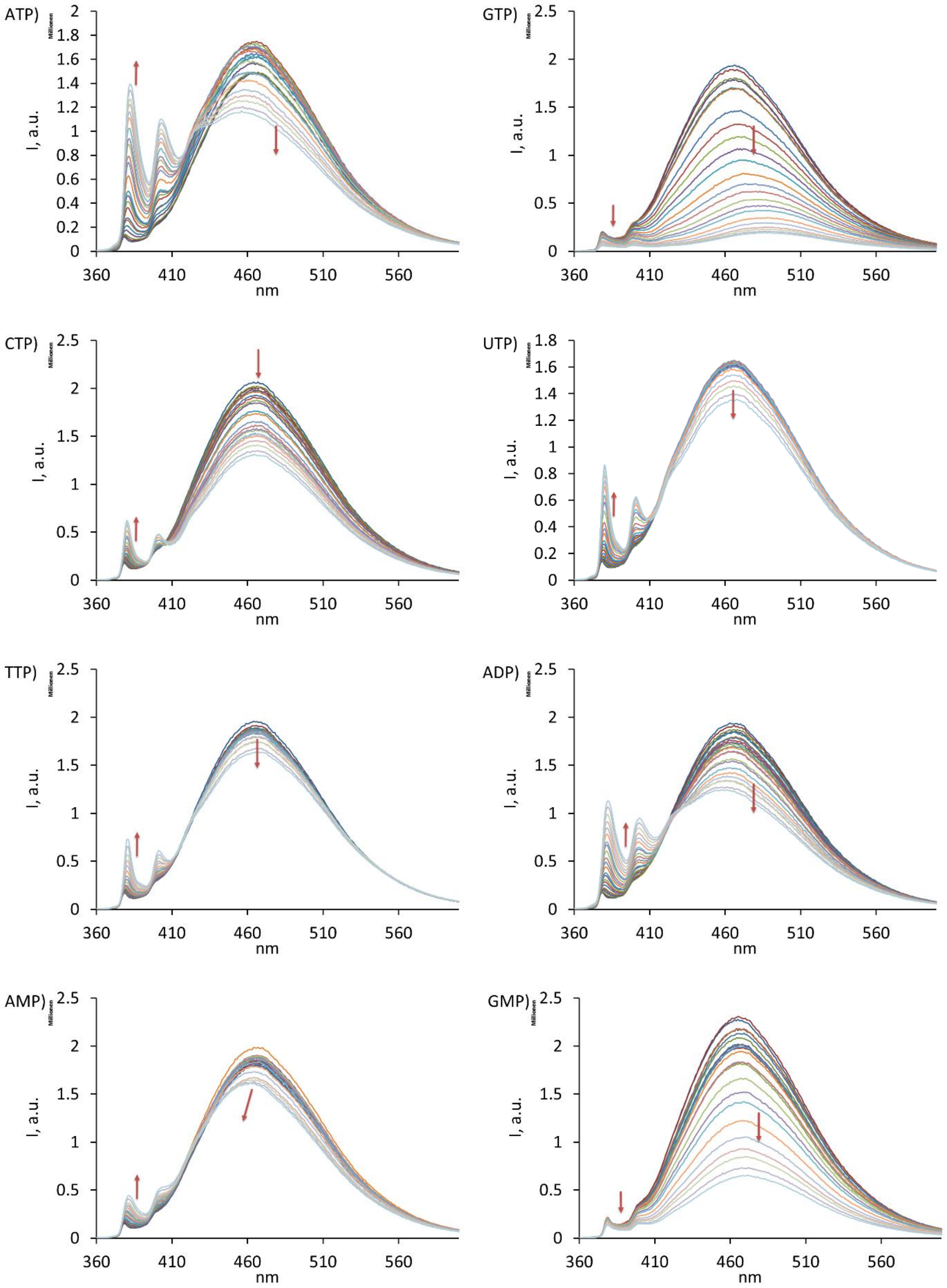

Figure S13. Fluorescence changes for **4** induced by addition of NTPs and other nucleoside mono- and diphosphates.

UV-Vis studies

UV-Vis titrations were carried out were carried out in the same manner as the fluorescence titrations. To a receptor solution with 10^{-5} M concentration, a solution of the receptor (10^{-5} M)and a nucleotide (0.02 M) was added in portions. At each step a spectrum was measured. All the spectra were combined by using HypSpec program and fitted to yield the binding constants.

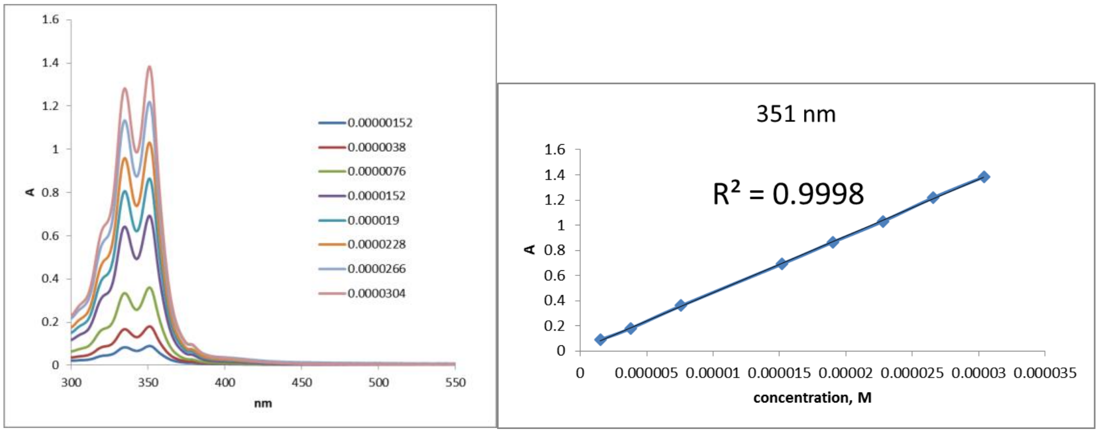

Figure S14. UV-Vis spectral changes upon dilution of receptor **1** with linear Lambert-Beer dependence, which indicates the absence of receptor aggregation in aqueous solution. Conditions: 50 mM MES buffer, pH 6.2, 6% DMSO.

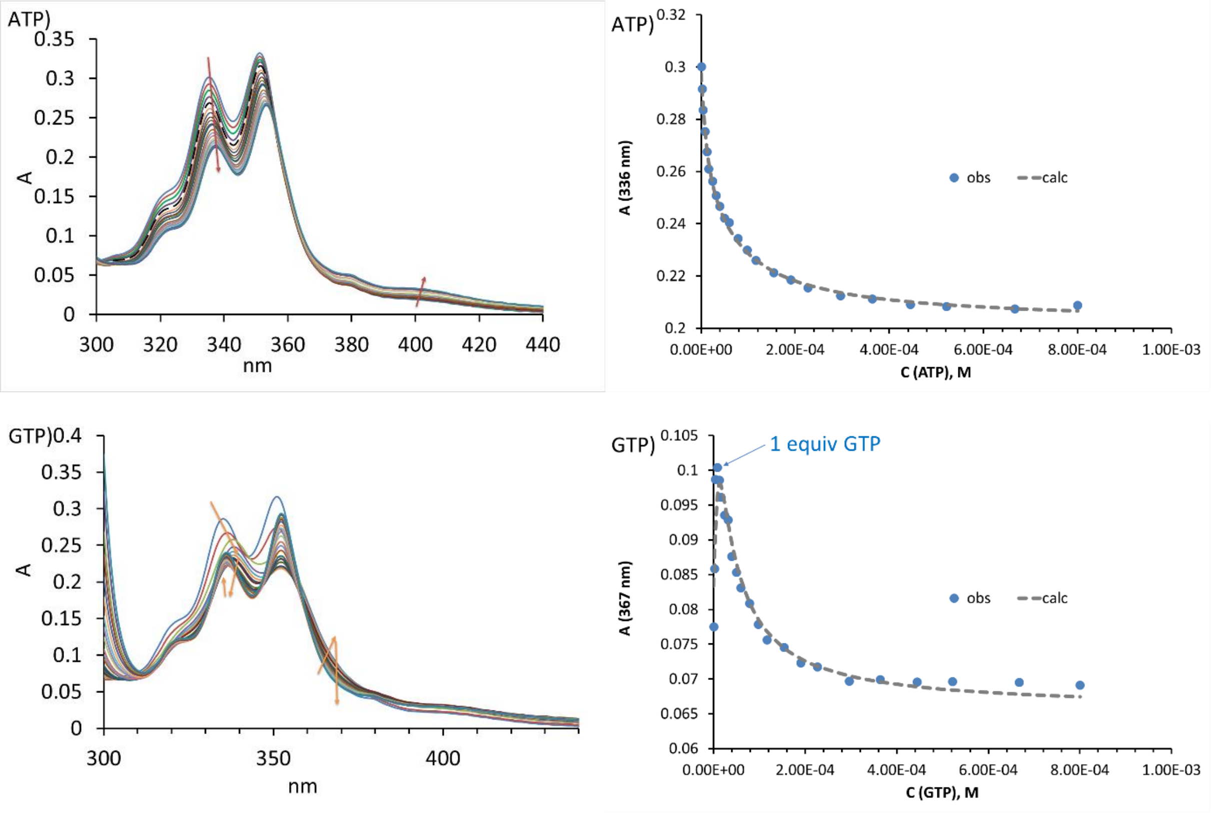

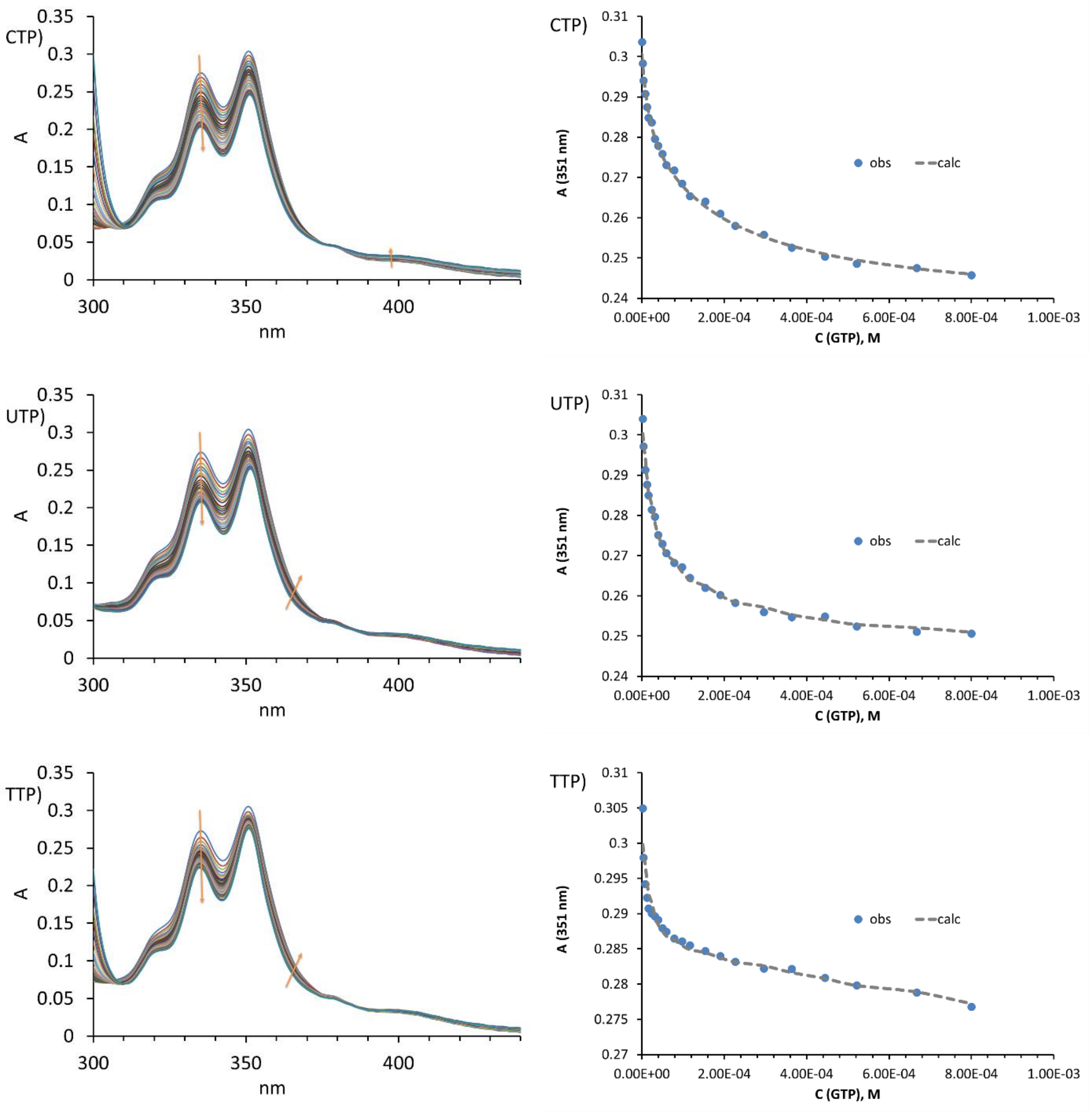

Figure S15. UV-Vis changes and fitting curves for receptor **1** with selected nucleotides. The calculated binding constants are:

ATP: logK_{11}=5.50, logK_{12}=4.12

GTP: logK_{11}=5.82, logK_{12}=4.43

CTP: logK_{11}=5.06, logK_{12}=3.71

UTP: logK_{11}=5.05, logK_{12}=3.82

TTP: logK_{11}=5.05, logK_{12}=3.82.

ITC titration of 1 with ATP.

The ITC experiments were evaluated by the help of the program SupraFit [C. Hübler, Institut of Organic Chemistry, TU Freiberg, Suprafit https://github.com/conradhuebler/SupraFit 02.2020].The calculated results are in good agreement with those obtained by the program NanoAnalyze™(TA Instruments). In the fitting average n values is 1.5.

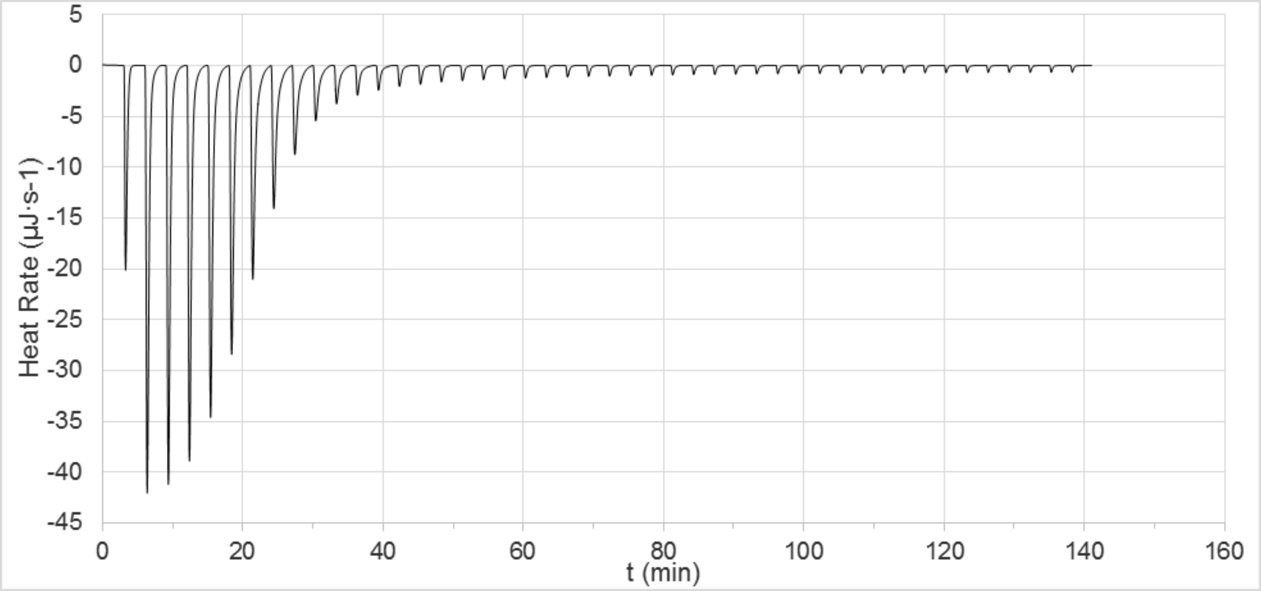

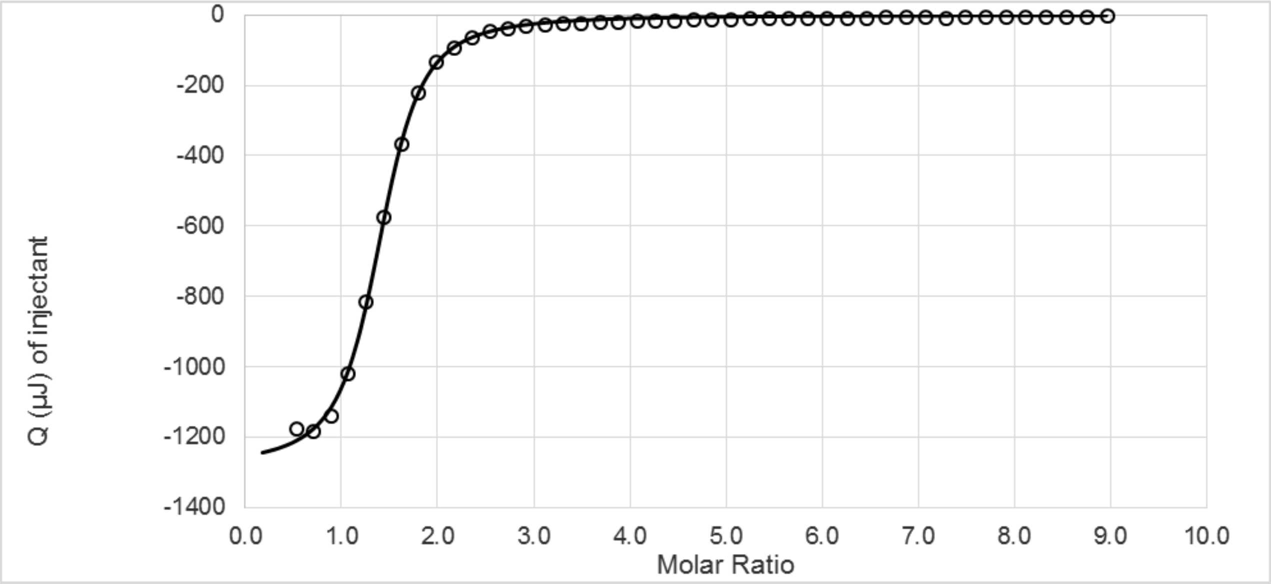

Figure S16. ITC titration: heat vs. time plots for addition of $Na_2C_2O_4$ and $NaClO_4$ together with fitting graphics.

DFT calculations

Coordinates of the adenosine complex with **1**H_4^{4+}

MOL> cartesian
MOL> 7 3.86129517 1.02124969 -2.87970200
MOL> 7 -1.46119457 6.40528557 -2.23717088

```
MOL>  1   -0.71887962   5.95847470  -1.67450691
MOL>  7    5.98506780   3.19240010  -3.24858700
MOL>  6   -2.53090664   5.40433428  -2.49617882
MOL>  1   -2.03204053   4.47646928  -2.78358311
MOL>  1   -3.13878767   5.74643859  -3.33867785
MOL>  6   -0.81802455   7.02925875  -3.45339495
MOL>  1   -1.56464618   7.69319217  -3.89233964
MOL>  1   -0.61041447   6.22781755  -4.15514255
MOL>  6    0.39453425   7.75274002  -2.98205453
MOL>  6    0.22983798   9.01047970  -2.41304783
MOL>  1   -0.75199481   9.47323072  -2.39747277
MOL>  6    1.30341102   9.69292294  -1.88528216
MOL>  1    1.16329472  10.67322259  -1.44612596
MOL>  6    2.57490616   9.13131610  -1.90861035
MOL>  6    2.76273942   7.86469842  -2.49353866
MOL>  6    1.66446518   7.16918812  -3.03311350
MOL>  6    1.91889401   5.89289999  -3.64832800
MOL>  1    1.09914057   5.32773947  -4.06743616
MOL>  6    3.15652833   5.39704633  -3.75619267
MOL>  1    3.29097173   4.45024670  -4.25705641
MOL>  6    4.30258347   6.09072531  -3.23076024
MOL>  6    5.61212482   5.61677076  -3.37163187
MOL>  6    6.66643074   6.30949078  -2.78774354
MOL>  1    7.68379156   5.94607624  -2.89826383
MOL>  6    6.44443409   7.47970787  -2.09232452
MOL>  6    5.16067423   8.00854087  -1.99573633
MOL>  6    4.07794676   7.30861391  -2.56292697
MOL>  6    3.70281522   9.82522722  -1.34034785
MOL>  1    3.53287876  10.79344045  -0.88306564
MOL>  6    4.92742113   9.28334601  -1.36267228
MOL>  1    5.77325074   9.80246820  -0.92370292
MOL>  6    5.91079937   4.39147424  -4.16289425
MOL>  1    5.15274842   4.17484917  -4.90837784
MOL>  1    6.87918325   4.45076271  -4.66203379
MOL>  6    5.92984062   1.87038161  -3.95867198
MOL>  1    6.49244191   1.15498397  -3.35486144
MOL>  1    6.43692521   1.98610286  -4.91936227
MOL>  6    4.48936261   1.41914337  -4.13037929
MOL>  1    3.92007747   2.23982038  -4.58188760
MOL>  1    4.46951675   0.58709826  -4.83983722
MOL>  6    4.05775728  -0.36651591  -2.49765095
MOL>  1    5.09839945  -0.71144193  -2.57893808
MOL>  1    3.46513761  -1.01208035  -3.15640550
MOL>  1    6.86154300   3.26356380  -2.72149822
MOL>  7   -2.67953382   4.92802506  -0.04587009
MOL>  7    2.23683132  -0.20030104  -0.80973331
MOL>  1    2.06640506   0.75571368  -1.14694751
MOL>  7   -4.23528246   4.02658603   2.07982316
MOL>  6    3.65671613  -0.57631948  -1.05535340
MOL>  1    4.26375178   0.04144732  -0.39912847
MOL>  1    3.77886507  -1.62361093  -0.76467675
MOL>  6    1.83137847  -0.28234088   0.65145361
MOL>  1    0.79021694  -0.59396213   0.65747304
MOL>  1    2.42637240  -1.08465325   1.08939394
MOL>  6    3.59988298   2.54598429   2.38269959
MOL>  6    3.34364247   1.39529400   1.67215858
MOL>  1    4.17754086   0.76186727   1.40074462
MOL>  6    2.04385213   1.01675612   1.35344888
MOL>  6    0.96998841   1.81584691   1.77140876
MOL>  6    1.22439894   2.95445388   2.56423241
MOL>  6    2.55054247   3.32398527   2.86066894
MOL>  6    2.80359488   4.45580953   3.71602719
MOL>  1    3.83498431   4.71779840   3.92675593
MOL>  6    1.79349874   5.14019161   4.26694038
MOL>  1    1.97817450   5.97790942   4.92927432
MOL>  6    0.42420903   4.78148458   3.99681498
MOL>  6   -0.62349194   5.46825879   4.60168407
MOL>  1   -0.40468916   6.30129671   5.26062016
MOL>  6   -1.92901457   5.07428051   4.39126534
MOL>  1   -2.72717353   5.60514426   4.90028211
MOL>  6   -2.22602775   4.00131251   3.55861627
MOL>  6   -1.19079261   3.31385523   2.91112004
MOL>  6    0.14417242   3.69882295   3.13833001
MOL>  6   -0.41057915   1.50017559   1.49101942
MOL>  1   -0.65201721   0.66352081   0.84988346
MOL>  6   -1.42014537   2.19760019   2.02864133
MOL>  1   -2.43198764   1.88227985   1.81509065
```

```
MOL>  6   -3.65090935  3.60056983  3.40604860
MOL>  1   -4.27263020  4.06680395  4.17087949
MOL>  1   -3.79940625  2.51842462  3.45057850
MOL>  6   -4.18755491  5.48678572  1.78187319
MOL>  1   -5.04274462  5.69907025  1.13484735
MOL>  1   -4.31455952  6.04729342  2.70956244
MOL>  6   -2.88669767  5.82736470  1.07053354
MOL>  1   -2.05382256  5.71496627  1.76241510
MOL>  1   -2.94577292  6.88967499  0.79524381
MOL>  6   -3.41017451  5.22239596 -1.27152765
MOL>  1   -4.12347020  4.42149436 -1.52019508
MOL>  1   -4.00153180  6.13263798 -1.13523938
MOL>  1   -5.21050362  3.69902875  2.05489551
MOL>  1   -1.70807991  4.67462449 -0.17838069
MOL>  1    5.21775717  3.25091550 -2.56538262
MOL>  1    1.64202033 -0.82836260 -1.36236345
MOL>  1   -1.84671296  7.17183965 -1.68241960
MOL>  1   -3.73270652  3.56605829  1.31412638
MOL>  1    7.27791045  8.01369952 -1.64800321
MOL>  1    4.62030253  2.81879985  2.62786674
MOL>  1    4.12431104  1.64900604 -2.12566603
MOL>  6    2.82378201  7.74182698  2.49183889
MOL>  6   -0.28579011  9.67416455  2.20929818
MOL>  6    1.16804110  9.29042081  2.17665939
MOL>  6    2.54744175  7.91304840  1.02247908
MOL>  6    1.48741027  8.12638789  3.06498081
MOL>  6    1.90140267  4.74779329 -0.61787992
MOL>  6    0.76464940  6.39900613  0.08545829
MOL>  6    2.78745415  5.63477104 -0.02010533
MOL>  6    2.46752804  3.61699577 -1.22733748
MOL>  6    4.53780773  4.37469182 -0.49485649
MOL>  1   -0.45637045 10.56713722  1.60059828
MOL>  1    3.76533121  8.82743358  3.78214606
MOL>  1   -0.88392310  8.86535831  1.77419940
MOL>  1    2.14303272  2.22019085 -2.65043065
MOL>  1    3.12593940  6.72027630  2.72296944
MOL>  1    1.77094292 10.14276991  2.48805749
MOL>  1    3.45974442  8.15610621  0.47951546
MOL>  1    0.76486915  7.32908960  2.87264152
MOL>  1    0.79801703  8.97067000  4.63411892
MOL>  1   -0.01711966  7.10282541  0.32780551
MOL>  1    0.74352568  2.97976100 -2.03663170
MOL>  1    5.60909967  4.17325508 -0.43399148
MOL>  7    2.04311562  6.68508482  0.41475364
MOL>  7    0.60473535  5.26353670 -0.54705384
MOL>  7    1.69014079  2.67425879 -1.86457979
MOL>  7    4.10431844  5.46948862  0.07767064
MOL>  7    3.79437944  3.46280972 -1.16708501
MOL>  8    3.78948514  8.65988912  2.83180494
MOL>  8    1.56171156  8.42306177  4.40221776
MOL>  8    1.57735940  8.85628764  0.90343207
MOL>  8   -0.67185814  9.84900986  3.55051441
MOL>  1   -0.91360730 10.76475382  3.73279973
MOL> Energy =  -2990.1296417286
```

X-ray studies

Single clear light colourless plate crystals of **19Kat_OSH01_2** recrystallised from a mixture of methanol and TCM by solvent layering. A suitable crystal with dimensions 0.39 × 0.25 × 0.11 mm^3 was selected and mounted on a mylar loop in perfluoroether oil on a SuperNova, Dual, Cu at home/near, Atlas diffractometer. The crystal was kept at a steady T = 152.95(10) K during data collection. The structure was solved with the **ShelXT** (Sheldrick, 2015) solution program using dual methods and by using **Olex2**[5] as the graphical interface. The model was refined with **ShelXL** 2018/3 (Sheldrick, 2015) using full matrix least squares minimisation on $\boldsymbol{F^2}$.

Crystal Data. $C_{48}H_{70}N_6O_8$, M_r = 859.10, monoclinic, $P2_1/c$ (No. 14), a = 17.8077(5) Å, b = 14.2917(4) Å, c = 8.9714(3) Å, β = 96.735(3)°, $\alpha = \gamma$ = 90°, V = 2267.49(12) Å^3, T = 152.95(10) K, Z = 2, Z' = 0.5, μ(Cu K_α) = 0.691, 6870 reflections measured, 3613 unique (R_{int} = 0.0228) which were used in all calculations. The final wR_2 was 0.2295 (all data) and R_1 was 0.0720 (I > 2(I)).

A clear light colourless plate-shaped crystal with dimensions 0.39 × 0.25 × 0.11 mm^3 was mounted on a mylar loop in perfluoroether oil. Data were collected using a SuperNova, Dual, Cu at home/near, Atlas diffractometer equipped with a Cryojet Oxford Instruments low-temperature device operating at T = 152.95(10) K.

Data were measured using ω scans using Cu K_α radiation. The diffraction pattern was indexed and the total number of runs and images was based on the strategy calculation from the program **CrysAlisPro** (Rigaku, V1.171.40.53, 2019). The maximum resolution that was achieved was Θ = 63.693° (0.86 Å).

The diffraction pattern was indexed and the total number of runs and images was based on the strategy calculation from the program **CrysAlisPro** (Rigaku, V1.171.40.53, 2019)The unit cell was refined using **CrysAlisPro** (Rigaku, V1.171.40.53, 2019) on 2494 reflections, 36% of the observed reflections.

Data reduction, scaling and absorption corrections were performed using **CrysAlisPro** (Rigaku, V1.171.40.53, 2019). The final completeness is 96.30 % out to 63.693° in Θ. A gaussian absorption correction was performed using CrysAlisPro 1.171.40.53 (Rigaku Oxford Diffraction, 2019) Numerical absorption correction based on gaussian integration over a multifaceted crystal model Empirical absorption correction using spherical harmonics, implemented in SCALE3 ABSPACK scaling algorithm.. The absorption coefficient μ of this material is 0.691 mm^{-1} at this wavelength (λ = 1.54184Å) and the minimum and maximum transmissions are 0.536 and 1.000.

The structure was solved and the space group $P2_1/c$ (# 14) determined by the ShelXT (Sheldrick, 2015) structure solution program using using dual methods and refined by full matrix least squares minimisation on $\boldsymbol{F}^2$ using version 2018/3 of ShelXL 2018/3 (Sheldrick, 2015). All non-hydrogen atoms were refined anisotropically. Hydrogen atom positions were calculated geometrically and refined using the riding model. Most hydrogen atom positions were calculated geometrically and refined using the riding model, but some hydrogen atoms were refined freely.

_diffrn_special_details: The asym unit contains two molecules of H2O and two molecules of MeOH

_exptl_absorpt_process_details: CrysAlisPro 1.171.40.53. Numerical absorption correction based on gaussian integration over a multifaceted crystal modelEmpirical absorption correction using spherical harmonicsas implemented in SCALE3 ABSPACK.

CCDC Deposition Number 1985471.

References

[1] M. V. Ivanov, K. Thakur, A. Boddeda, D. N. Wang, R. Rathore, *J Phys Chem C* **2017**, *121*, 9202-9208.

[2] Y. Niko, S. Sasaki, K. Narushima, D. K. Sharma, M. Vacha, G.-i. Konishi, *J Org Chem* **2015**, *80*, 10794-10805.

[3] L. Fabbrizzi, M. Licchelli, N. Marcotte, F. Stomeo, A. Taglietti, *Supramol Chem* **2002**, *14*, 127-132.

[4] P. Gans, A. Sabatini, A. Vacca, *Talanta* **1996**, *43*, 1739-1753.

[5] O. V. Dolomanov, L. J. Bourhis, R. J. Gildea, J. A. K. Howard, H. Puschmann, *J Appl Crystallogr* **2009**, *42*, 339-341.
